# A Microtubule‐Associated Protein Functions in Preventing Oocytes from Evading the Spindle Assembly Checkpoint

**DOI:** 10.1002/advs.202413097

**Published:** 2024-12-25

**Authors:** Changyin Zhou, Xue Zhang, Genlu Xu, Yuting Ran, Hui Wang, Xuefeng Xie, Ang Li, Fei Li, Xiaozhen Li, Jinlong Ding, Mianqun Zhang, Qing‐Yuan Sun, Xiang‐Hong Ou

**Affiliations:** ^1^ Guangzhou Key Laboratory of Metabolic Diseases and Reproductive Health Guangdong‐Hong Kong Metabolism & Reproduction Joint Laboratory Reproductive Medicine Center The Affiliated Guangdong Second Provincial General Hospital of Jinan University Guangzhou 510317 China; ^2^ School of Biomedical and Pharmaceutical Sciences Guangdong University of Technology Guangzhou 510006 China; ^3^ College of Animal Science and Technology Anhui Agricultural University Key Laboratory of Local Livestock and Poultry Genetical Resource Conservation and Breeding of Anhui Province Hefei 230036 China

**Keywords:** aneuploid eggs, microtubule‐associated protein, oocyte meiosis, spindle assembly checkpoint

## Abstract

Aneuploidy eggs are a common cause of human infertility, spontaneous abortion, or trisomy syndromes. The spindle assembly checkpoint (SAC) plays a crucial role in preventing aneuploidy in oocytes, yet it is unclear if additional mechanisms exist to ensure oocyte adherence to this checkpoint. It is now revealed that the microtubule‐associated protein NUSAP can prevent oocytes from evading the SAC and regulate the speed of the cell cycle. Mechanistically, the study identifies NUSAP as a novel stabilizer of the E3 ubiquitin ligase APC/C^CDH1^, protecting CDH1 from SCF^BTRC^‐mediated degradation. Depletion of NUSAP reduces CDH1 protein level, leading to abnormal spindle assembly and chromosome alignment, and disrupting the balance of cell cycle proteins. This misregulated balance causes oocytes to evade the SAC. Consequently, these abnormal oocytes not only fail to arrest at metaphase but also accelerate the cell process, ultimately resulting in the production of aneuploid eggs. Together, the findings not only clarify the existence of mechanisms that ensure oocytes compliance with the spindle assembly checkpoint but also expand the new functions of NUSAP beyond its role as a microtubule‐ associated protein.

## Graphical abstract

Aneuploidy eggs are a common cause of human infertility, spontaneous abortion, or trisomy syndromes. The spindle assembly checkpoint (SAC) plays a crucial role in preventing aneuploidy in oocytes, yet it is unclear if additional mechanisms exist to ensure oocyte adherence to this checkpoint. It is now revealed that a microtubule‐associated protein can prevent oocytes from evading the SAC.

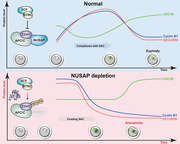

## 1 Introduction

Microtubule‐associated proteins have been recognized for their ability to bind to microtubules, providing them with increased stability. They serve as essential orchestrators within the microtubule cytoskeleton, contributing significantly to a range of cellular functions. Microtubule‐associated proteins are instrumental in the organization of spindle fibers, the facilitation of intracellular transport mechanisms, the progression of neuronal maturation, and the establishment of the ciliary axoneme.^[^ ^1^ ^]^

NUSAP is a conserved microtubule‐associated protein in vertebrates, and it bundles microtubules and links them to chromosomes, playing a crucial role in chromosome‐dependent spindle assembly.^[^ ^2^ ^]^ Previous studies indicated that NUSAP functions as a mitotic Ran GTPase target that stabilizes and crosslinks microtubules.^[^ ^3^ ^]^ NUSAP is selectively expressed in proliferating cells and highly transcribed and expressed in late S/G2 phase of the cycle with a very low expression level in G1 phase.^[^ ^2a^ ^]^ In interphase, NUSAP is confined to the nucleus and concentrated in nucleoli. During prometaphase, NUSAP underwent a redistribution process, moving away from the nucleoli and relocating near the chromosomes. This results in the formation of bundles, which gradually become more distinct as metaphase and early anaphase progress. These NUSAP bundles colocalized with microtubules of the central spindle.^[^ ^2^ ^]^

Suppression of NUSAP results in delayed entry into mitosis, defective cytokinesis, and short spindle.^[^ ^2^, ^4^ ^]^ NUSAP deficiency in mice leads to early embryonic lethality, sustained spindle checkpoint activity, and mitotic arrest, eventually leading to caspase activation and apoptotic cell death.^[^ ^5^ ^]^ NUSAP also plays a pivotal role in chromosome oscillation,^[^ ^6^ ^]^ and it is upregulated in several types of cancer.^[^ ^7^ ^]^

In the present study, we investigated the subcellular distribution, expression, and function of the microtubule‐associated protein NUSAP during female meiosis I in mice. We reveal the unprecedented role of NUSAP in preventing oocytes from evading the SAC, maintaining proper cell cycle speed, and ultimately ensuring oocyte euploidy. Depletion of NUSAP results in SCF^BTRC^‐mediated degradation of CDH1, resulting in abnormal spindle assembly, chromosome misalignment, and an altered balance of cell cycle proteins. Although the deletion of NUSAP does not affect the localization of SAC proteins to kinetochores, the abnormal abundance of cell cycle proteins causes oocytes to evade SAC surveillance, rendering the SAC in name only. This results in oocytes with abnormal spindles and misaligned chromosomes not only failing to be arrested at metaphase, but also accelerating the meiotic speed and ultimately resulting in the production of aneuploid eggs.

## 2 Results

### 2.1 Subcellular Localization and Expression of NUSAP During Oocyte Meiosis

**Figure 1:**
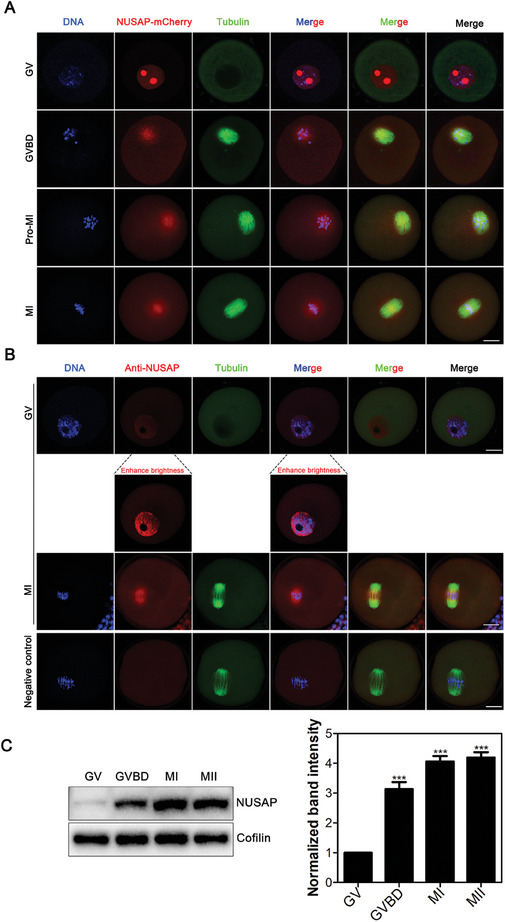
Expression dynamics of NUSAP in mouse meiotic oocytes. A) Representative images of NUSAP‐mCherry localization during mouse oocyte meiosis. Mouse oocytes were microinjected with NUSAP‐mCherry mRNA at GV stage, maintained for 2 hours in 50 µm IBMX before being washed into IBMX‐free medium to allow development to GVBD (2 hours post‐IBMX release), Pro‐M I (6 hours post‐IBMX release) and M I (8 hours post‐IBMX release), respectively, followed by DNA staining with Hoechst and immunostaining with α‐tubulin antibody. Scale bar, 20 µm. B) Representative images of NUSAP localization in oocytes. Mouse oocytes were immunostained with NUSAP and α‐tubulin antibodies. Negative control refers to the absence of the NUSAP‐specific primary antibody. Scale bar, 20 µm. C) Protein levels of NUSAP during oocyte meiosis were examined at the GV (Germinal Vesicle, no cultivation needed), GVBD (0 hours post‐GVBD), MI (6 hours post‐GVBD), and MII (10 hours post‐GVBD) stages using immunoblotting analysis. The blots were probed with anti‐NUSAP antibody and anti‐Cofilin antibody, respectively. The band intensity of NUSAP was normalized with that of Cofilin. ***p < 0.001.

To show the localization at different developmental stages of mouse oocyte maturation. We microinjected NUSAP‐mCherry mRNA into the germinal vesicle (GV) oocytes, followed by culturing to different developmental stages. Immunofluorescence and confocal imaging documented that NUSAP was predominantly present in the nucleus of GV oocytes, especially in the nucleolus (**Figure** **1**A). This is similar to the localization of NUSAP during the interphase of the cell cycle in somatic cells.^[^ ^2^ ^]^ To rule out the possibility that this localization pattern might be due to non‐physiological localization caused by an extremely large amount of overexpressed NUSAP, we injected a sufficiently low concentration of NUSAP‐mCherry to approximate the protein level of endogenous NUSAP (Figure S1B, Supporting Information), and found that the localization was consistent with our previous findings (Figure S1C, Supporting Information). Additionally, we observed similar localization upon injecting NUSAP‐mCherry into NUSAP‐depleted oocytes (Figure S1D, Supporting Information). After germinal vesicle breakdown (GVBD, that is, the resumption of meiosis), NUSAP overlapped significantly with microtubules and chromosomes (Figure 1A; Figure S1A, Supporting Information), similar to that in somatic cells.^[^ ^2^ ^]^

Subsequently, we used a NUSAP antibody to examine the localization of NUSAP, and the results showed that the localization was essentially consistent with that of NUSAP‐mCherry (Figure 1B). The difference was that the antibody did not reveal the presence of NUSAP in the nucleolus of GV, possibly because the nucleolus is a complex and highly dense cellular structure,^[^ ^8^ ^]^ and oocytes are special, being the largest cells in volume, and the germinal vesicle is a unique structure (and also larger in volume compared to the nuclei of somatic cells), these combined factors may make it difficult for some antibodies to effectively label the nucleolus of oocytes. Next, we treated oocytes with nocodazole to depolymerize microtubules and observe the localization of NUSAP, and we found that NUSAP was still localized on chromosomes (Figure S1E, Supporting Information). This may suggest that the localization of NUSAP on chromosomes is not dependent on microtubules.

We next assessed the protein expression level of NUSAP during oocyte meiosis. Oocytes collected from GV, GVBD, metaphase I (M I) and metaphase II (M II) stages were subjected to immunoblotting analysis using anti‐NUSAP antibody, and the results show that NUSAP has lower expression in GV oocytes, with a significant increase in protein levels in GVBD stage compared to the GV phase. There is a slight increase in protein levels in the M I phase compared to the GVBD stage, and this high level is essentially maintained during the MII phase (Figure 1C). This was essentially consistent with the results of Proteomics data^[^ ^9^ ^]^ (Figure S1F, Supporting Information). The high expression of NUSAP during oocyte meiosis I may indicate that it plays a critical role in the regulation of oocyte meiotic maturation.

### 2.2 Depletion of NUSAP Accelerates Meiotic Progression

**Figure 2:**
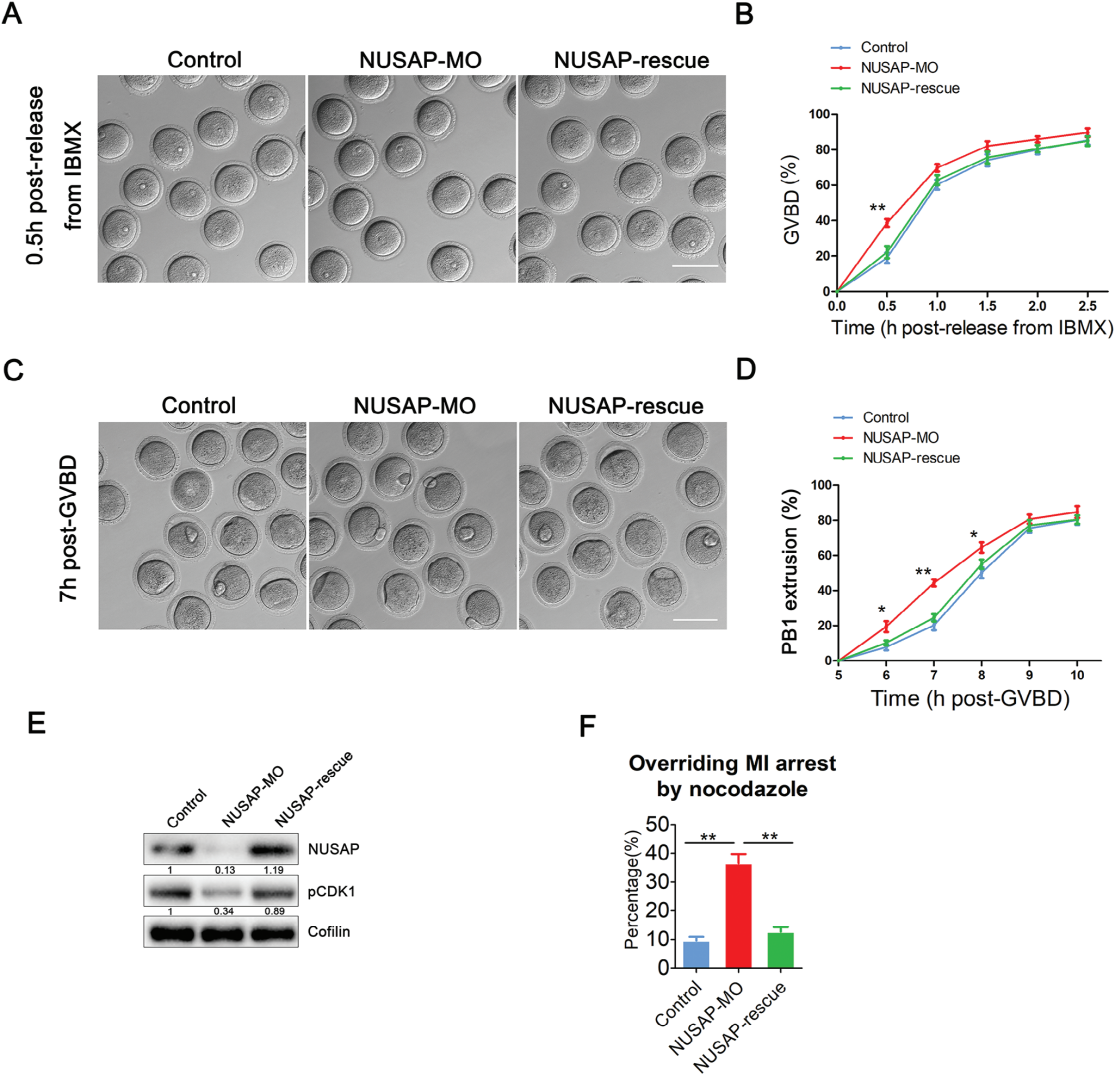
Effect of NUSAP depletion on meiotic progression in mouse oocytes. A) Representative images of the occurrence of GVBD in control, NUSAP‐MO and NUSAP‐rescue (NUSAP‐MO + NUSAP‐cRNA) oocytes at 0.5 hours following release from IBMX. Scale bar, 100 µm. B) The incidences of GVBD at 0.5, 1, 1.5, 2 and 2.5 hours post‐IBMX release were quantified in control (n = 116), NUSAP‐MO (n = 106) and NUSAP‐rescue (n = 118) oocytes. For the rescue experiment, GV oocytes were injected with NUSAP‐specific morpholino oligonucleotides and maintained for 20 hours in 50 µm IBMX before being injected with morpholino‐resistant NUSAP mRNA and maintained for a further 2 hours in 200 µm IBMX to allow time for NUSAP translation. Oocytes were then washed into IBMX‐free medium to allow resumption of meiosis. C) Representative images of PBE in control, NUSAP‐MO and NUSAP‐rescued oocytes at the time point of 7 hours post‐GVBD. Scale bar, 100 µm. D) Quantitative analysis of PBE rates was shown in control (n = 130), NUSAP‐MO (n = 124) and NUSAP‐rescue (n = 118) oocytes at consecutive time points of post‐GVBD. E) Protein levels of pCDK1 were assessed by immunoblots in control, NUSAP‐MO and NUSAP‐rescue oocytes at GV stage. The blots were probed with NUSAP, pCDK1 and Cofilin antibodies. F) The proportion of oocytes overriding metaphase I arrest following nocodazole treatment was recorded in control (n = 105), NUSAP‐MO (n = 104) and NUSAP‐rescue (n = 102) oocytes. Oocytes injected with the indicated morpholino and/or mRNA were cultured with 400 nm nocodazole from 4 h post‐GVBD and the PBE rate was scored at 10 h post‐GVBD. Data of (B), (D), and (F) were presented as mean value (mean ± SEM) of at least three independent biological replicates. *p < 0.05, **p < 0.01.

To investigate the function of NUSAP, we used morpholino oligonucleotides (MO) to reduce its protein level in oocytes. In order to assess the effect of NUSAP depletion on the meiotic progression, we recorded the frequency of GVBD and PBE (polar body extrusion), two critical developmental events during oocyte meiosis. Surprisingly, depletion of NUSAP accelerated not only the process of GVBD but also the PBE process (**Figure** **2**A–D). At 0.5 hours of post release from IBMX, the GVBD rate was significantly higher in the knockdown group (Figure 2A,B). A considerably higher incidence of PBE was observed in NUSAP‐depleted oocytes than in control oocytes at the time point of 6 to 8 hours post‐GVBD (Figure 2C,D), although the final PBE rate at 10 hours post‐GVBD was comparable between two groups (Figure 2D). To rule out the possibility that the acceleration of cell cycle processes was due to off‐target effects of the morpholino oligonucleotides, we expressed the exogenous NUSAP in NUSAP‐depleted oocytes to monitor the meiotic progression. As anticipated, in the rescued oocytes, the frequency of GVBD or PBE was reduced to the level indistinguishable from controls (Figure 2B,D). This acceleration of meiotic progression was reproduced by knockdown of NUSAP with its targeted siRNA (Figure S3A–D, Supporting Information). We repeated the experiments using siRNA that can efficiently target knockdown of NUSAP (Figure S2, Supporting Information), which recapitulates this acceleration of meiotic progression (Figure S3, Supporting Information).

Accelerated GVBD is often associated with elevated CDK1 kinase activity. We subsequently examined the level of inhibitory phosphorylation of CDK1 at site Tyr15. We found that the level of phosphorylation of CDK1 in NUSAP‐MO oocytes was significantly decreased and that this reduction was rescued in the NUSAP‐rescue oocytes (Figure 2E). This finding can be further confirmed by the results of NUSAP‐siRNA experiments (Figure S2B, Supporting Information). We conducted experiments utilizing RO‐3306, a specific inhibitor of CDK1 kinase activity, and discovered that it was indeed capable of rescuing the GVBD kinetics in NUSAP‐depleted oocytes (Figure S3E, Supporting Information). This suggests that the acceleration of GVBD caused by NUSAP depletion is associated with elevated CDK1 activity. An abnormal SAC function often leads to premature PBE, which implies that SAC activity may be compromised or that oocytes may evade SAC monitoring. To further confirm the SAC control by NUSAP, we tested whether oocytes depleted of NUSAP would override the metaphase I arrest induced by nocodazole treatment. Nocodazole treatment results in partial depolymerization and instability of spindle microtubules, which activates the SAC and causes the oocytes to be arrested at M I stage, preventing their development to anaphase stage. However, if the SAC function in oocytes is abnormal or if they can evade SAC surveillance, a high proportion of oocytes will override the M I arrest and extrude the polar body. Thus, after nocodazole treatment, the fact that NUSAP‐deficient oocytes override the M I arrest may suggests that the SAC function in these oocytes is abnormal or that they can evade SAC surveillance (Figure 2F). Restoring NUSAP levels in NUSAP‐deficient oocytes leads to an arrest at the M I stage (Figure 2F), indicating that the rescued oocytes have regained normal SAC function or that they cannot evade SAC surveillance. To rule out the possibility that the loss of NUSAP counteracts the effect of nocodazole on microtubule stability, we analyzed the spindle morphology of NUSAP‐depleted oocytes after nocodazole treatment and quantified the number of kinetochores not attached to microtubules, finding no significant difference compared to control oocytes (Figure S4D,E, Supporting Information).

### 2.3 Depletion of NUSAP Causes Meiotic Spindle Defects and Chromosome Misalignment

**Figure 3:**
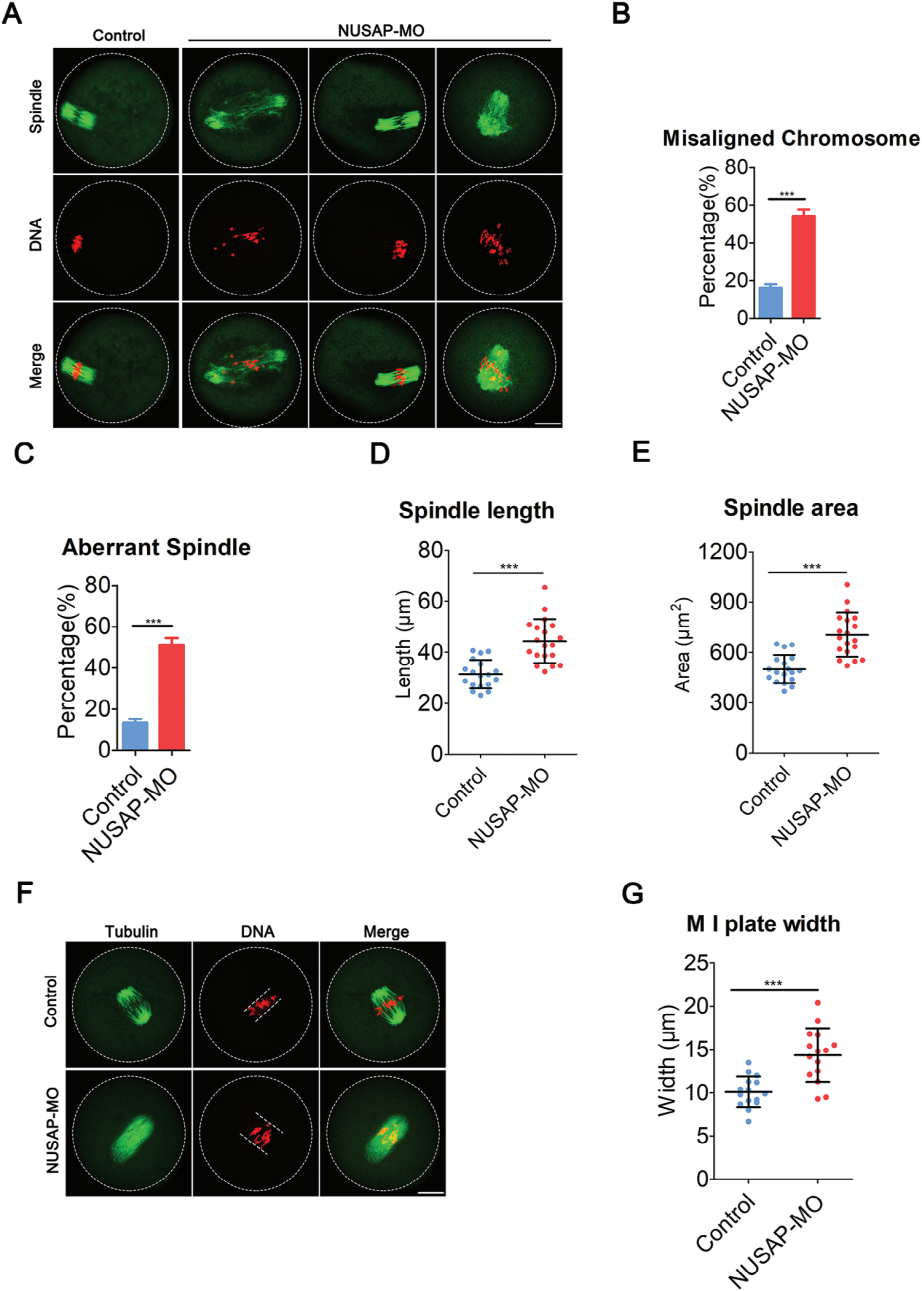
NUSAP is indispensable for meiotic spindle assembly and chromosome alignment. A) Representative images of spindle morphologies and chromosome alignment in control and NUSAP‐MO oocytes. At 6 hours post‐GVBD, oocytes were fixed and immunostained for α‐tubulin and DNA (PI). Scale bar, 20 µm. B) The rates of misaligned chromosomes were recorded in control (n = 74) and NUSAP‐MO (n = 68) oocytes. C) The rates of abnormal spindles were recorded in control (n = 74) and NUSAP‐MO (n = 68) oocytes. D,E) The spindle length and area were measured in control (n = 18) and NUSAP‐MO (n = 19) oocytes at 6 hours post‐GVBD. F) Representative images of the width of M I plate in control and NUSAP‐MO oocytes. At 6 hours after GVBD, oocytes were fixed and immunostained for α‐tubulin and DNA (PI). Scale bar, 20 µm. G) The width of M I plate was measured in control (n = 15) and NUSAP‐MO (n = 15) oocytes. Data of (B) and (C) were presented as mean percentage (mean ± SEM) of at least three independent biological replicates. Data of (D), (E) and (G) were presented as or mean value (mean ± SD) of at least three independent biological replicates. ***p < 0.001.

Given that the SAC acts as a monitor for spindle assembly and chromosome alignment, we subsequently examined the impact of NUSAP deficiency on spindle assembly and chromosome alignment. To this end, mouse oocytes from control and NUSAP‐depleted groups were immunolabeled with anti‐α‐tubulin antibody to visualize the spindle morphologies and counterstained with PI to observe the chromosome alignment. By striking contrast, control oocytes at M I stage usually show a typical barrel‐shaped spindle and well‐aligned chromosomes at the equator (**Figure** **3**A), we found a significant increase in the percentage of spindle defects and chromosome misalignment in NUSAP‐depleted oocytes (Figure 3A–C; Figure S4A–C, Supporting Information), which does not meet the control standards. Additionally, we restored the expression of NUSAP in NUSAP‐depleted oocytes and found that it significantly rescued the spindle assembly defects and chromosome misalignment caused by NUSAP deficiency (Figure S5C,D, Supporting Information). The spindle length and area in oocytes at M I stage were considerably increased in NUSAP‐depleted oocytes (Figure 3D,E). In addition, we measured the width of chromosome plate at M I stage and found it to be remarkably wider in NUSAP‐depleted oocytes than the controls (Figure 3F,G; Figure S5A,B, Supporting Information).

### 2.4 NUSAP Maintains the Stability of K‐MT Attachments and Prevents the Incidence of Aneuploidy in Oocytes

**Figure 4:**
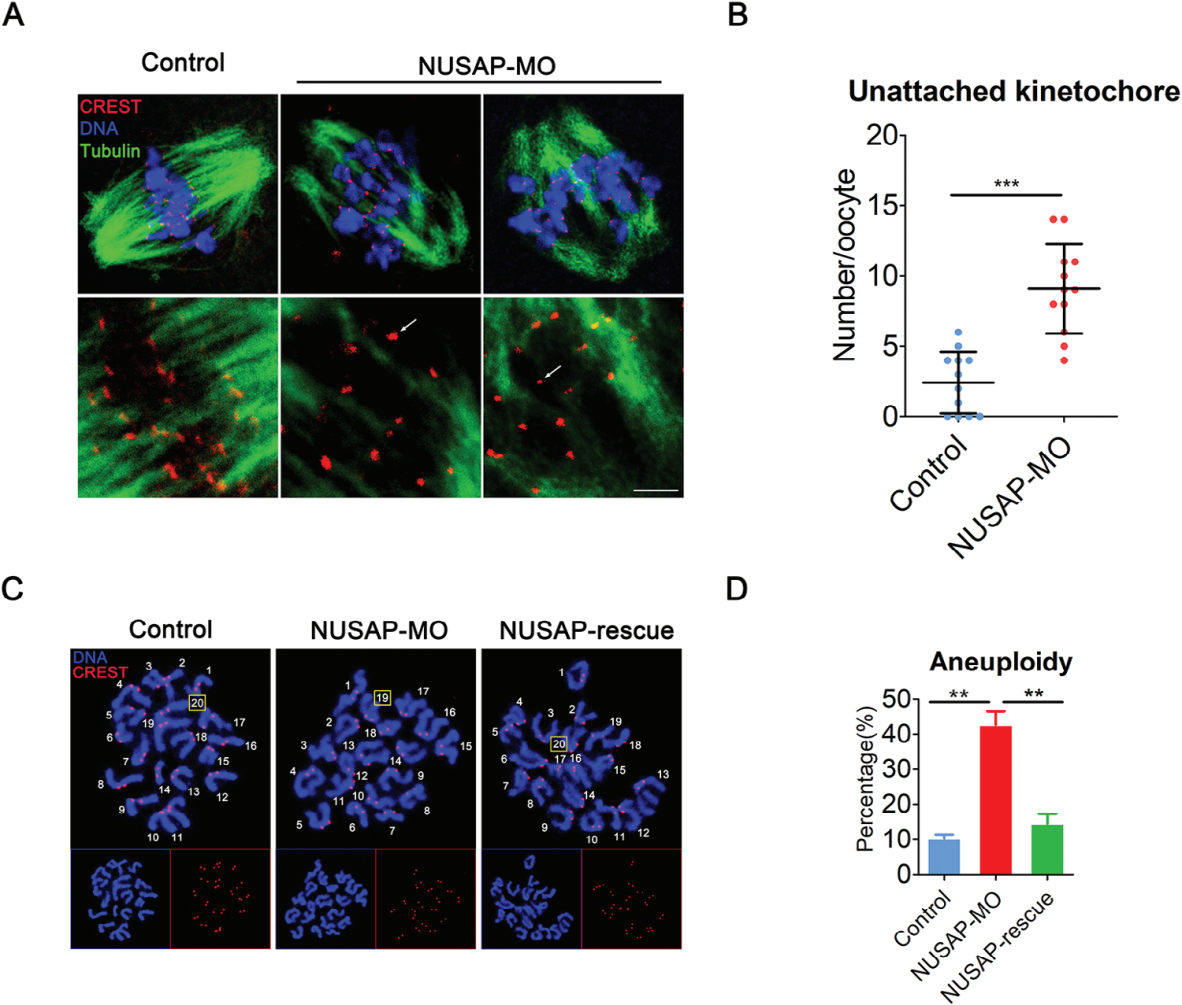
Depletion of NUSAP compromises K‐M attachments and generates aneuploidy in mouse oocytes. A) Representative images of K‐MT attachment in control and NUSAP‐MO oocytes. At 6 hours after GVBD, oocytes were incubated in M2 medium at 4 °C for 10 min to induce the depolymerization of unstable microtubules and then immediately fixed and immunostained for α‐tubulin, CREST, and DNA (Hoechst). White arrows indicate nonconnected kinetochores. Scale bar, 2.5 µm. B) The number of unattached kinetochores was recorded in control (n = 12) and NUSAP‐MO (n = 12) oocytes. C) Representative images of euploid and aneuploid M II eggs. Chromosome spreading was performed to count the number of chromosomes in control, NUSAP‐MO and NUSAP‐rescue oocytes at 10 hours after GVBD. The total number of univalents is indicated by the yellow square. Scale bar, 5 µm. D) The rates of aneuploid eggs were recorded in control (n = 30), NUSAP‐MO (n = 33) and NUSAP‐rescue (n = 27) oocytes. Data of (D) were presented as mean percentage (mean ± SEM) of at least three independent experiments. Data of (B) were presented as mean value (mean ± SD) of at least three independent experiments. **p < 0.01, ***p < 0.001.

The dramatically higher occurrence of misaligned chromosomes in NUSAP‐depleted oocytes implies that the K‐MT attachments might be impaired. To test it, oocytes were exposed to cold treatment for inducing depolymerization of microtubules that are not attached to kinetochores. We found that microtubules captured the kinetochores on the aligned bivalents at the equator in most of control oocytes (**Figure** **4**A,B); On the contrary, in NUSAP‐depleted oocytes, there was a higher frequency of kinetochores that were unattached to microtubules, with very few cold‐stable microtubules present (Figure 4A,B), suggesting that NUSAP is required for proper K‐MT attachments in meiosis.

Incorrect or instable K‐MT attachments would inevitably result in the establishment of unstable chromosome biorientation, which causes aneuploidy in mammalian eggs.^[^ ^10^ ^]^ To ask if the loss of NUSAP would generate aneuploidy in oocytes, karyotypic analysis of M II eggs was performed by chromosome spreading. We found that a large proportion of control oocytes had correct number of univalents to maintain the euploidy (Figure 4C). However, the incidence of aneuploid eggs that had more or less 20 univalents in NUSAP‐depleted oocytes was dramatically increased compared to that in controls (Figure 4D), suggesting that NUSAP is required to protect oocytes from aneuploidy. Consistently, in the rescue oocytes, the rate of aneuploidy was reduced to the normal level that was comparable to controls (Figure 4D). Similarly, depletion of NUSAP in oocytes by targeting siRNA resulted in aneuploidy (Figure S6, Supporting Information).

### 2.5 NUSAP Interacts with CDH1 and Maintains Its Protein Level to Prevent Oocytes from Evading the Spindle Assembly Checkpoint

**Figure 5:**
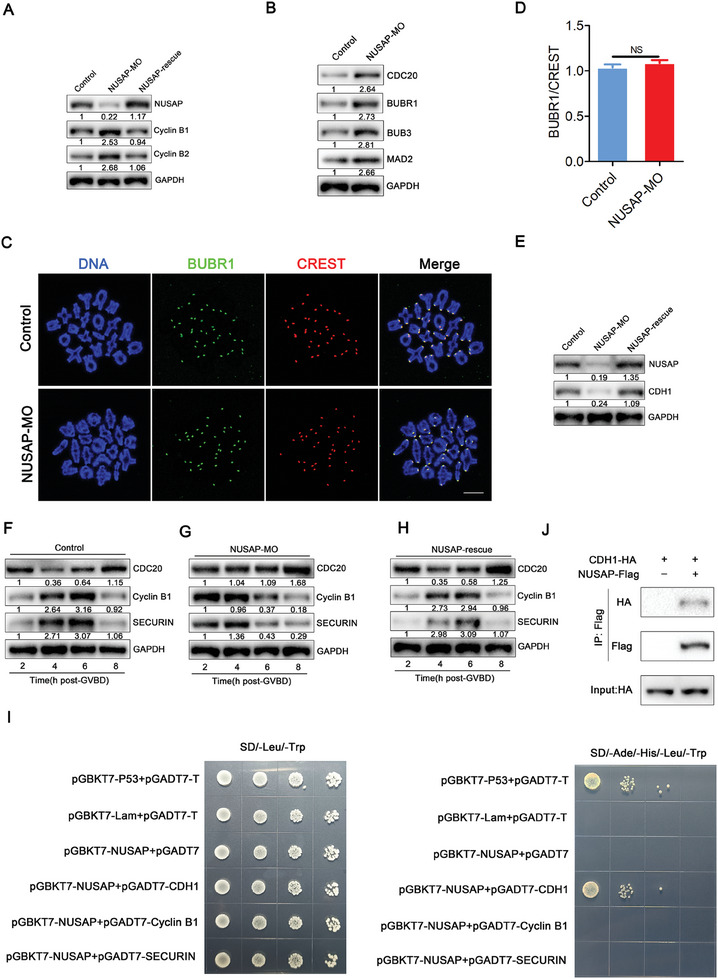
NUSAP stabilizes CDH1 protein to correctly regulate cell cycle protein abundance. A) Protein levels of Cyclin B1 and Cyclin B2 were assessed by immunoblots in control, NUSAP‐MO, and NUSAP‐rescue oocytes at GV stage. The blots were probed with NUSAP, Cyclin B1, Cyclin B2, and GAPDH antibodies. B) Protein levels of MCC were assessed by immunoblots in control and NUSAP‐depleted oocytes at GV stage. The blots were probed with CDC20, BUBR1, BUB3, MAD2 and GAPDH antibodies. C) Localization of BUBR1 at prometaphase I stage in control and NUSAP‐depleted oocytes. At 3 hours after GVBD, oocytes were fixed and immunostained for BUBR1, CREST and DNA (Hoechst). Scale bar, 10 µm. D) The relative fluorescence intensities of BUBR1 to CREST were measured in control (n = 200, kinetochores) and NUSAP‐depleted (n = 200, kinetochores) oocytes. The signal intensity of BUBR1 was normalized with that of CREST. E) Protein levels of CDH1 were assessed by immunoblots in control, NUSAP‐MO, and NUSAP‐rescue oocytes at GV stage. F–H) Western blot analysis of endogenous CDC20, Cyclin B1 and SECURIN levels at the times indicated after GVBD. In (F), the control group, immunoblots show that the early loss in CDC20 at 4 hours after GVBD, and Cyclin B1 or SECURIN decreased significantly after 6 hours of GVBD. In (G), the NUSAP‐MO group, immunoblots show that NUSAP depletion abolished CDC20 loss and brought forward Cyclin B1 and SECURIN degradation. In H, the NUSAP‐rescue group, the immunoblots showed a dynamic pattern of protein levels for CDC20, Cyclin B1, and SECURIN that was similar to that in the control group. I) Yeast two‐hybrid assay showing interaction of NUSAP with CDH1, Cyclin B1 or SECURIN. Yeast transformants expressing the plasmids harboring the indicated constructs were assayed for growth on selective plates (SD/‐Leu/‐Trp/‐His/‐Ade) and nonselective pates (SD/‐Leu/‐Trp). Physical interaction between NUSAP and CDH1 was identified in the test. The interaction of pGBKT7‐53 and pGADT7‐T was used as a positive control, whereas that of pGBKT7‐Lam and pGADT7‐T was used as a negative control. The BD‐NUSAP+AD was used as a negative control to exclude self‐activation of NUSAP. All transformants were assayed at specified concentrations of 1 × 10^6^, 1 × 10^5^, 1 × 10^4^, and 1 × 10^3^cells per one droplet. J) Co‐immunoprecipitation result showing NUSAP interaction with CDH1 in oocytes. 300 mouse oocytes were microinjected with NUSAP‐Flag and CDH1‐HA cRNA together or CDH1‐HA cRNA alone, maintained for a further 4 hours in 200µm IBMX to allow time for translation. Target proteins were immunoprecipitated using anti‐Flag beads and subjected to western blotting with Flag and HA antibodies. Input oocyte lysates were immunoblotted with anti‐HA antibody to determine the expression of CDH1. Data of (D) were presented as mean percentage (mean ± SEM) of at least three independent experiments.

Elevated CDK1 kinase activity often leads to acceleration of GVBD. We further explored the mechanisms leading to increased CDK1 kinase activity. Our studies and others have shown that CDK1 is activated by the binding of its regulatory subunits, Cyclin B1 or Cyclin B2.^[^ ^11^ ^]^ Hence, we evaluated the protein levels of Cyclin B1 and Cyclin B2 in NUSAP‐depleted oocytes via immunoblotting and found both to be elevated compared to control oocytes, and these elevations were rescued to normal levels in the NUSAP‐rescue oocytes (**Figure** **5**A).

Considering that NUSAP knockdown compromises the SAC mechanism, we subsequently examined the protein levels of BUBR1, BUB3, MAD2, and CDC20, the components of mitotic checkpoint complex (MCC) in oocytes. The results showed that their protein levels were all increased (Figure 5B). However, mRNA levels of these cell cycle proteins were not affected by NUSAP deletion (Figure S7A–F, Supporting Information), suggesting that it may affect their protein stability or efficiency of translation. In addition, BUBR1, a crucial part of the SAC complex, was immunostaining as an indicator.^[^ ^12^ ^]^ As shown in Figure 5C,D, BUBR1 was recruited to the unattached kinetochores at prometaphase I stage in control and NUSAP‐deficient oocytes, indicating that NUSAP deletion did not affect its recruitment to kinetochore. Additionally, we observed the localization of multiple SAC proteins at 5 hours post‐GVBD, and the statistics showed that none of them were affected by the depletion of NUSAP (Figure S8, Supporting Information).

The advancement of the cell cycle is facilitated by a protein degradation system referred to as the Anaphase Promoting Complex/Cyclosome (APC/C), which collaborates with either CDC20 or CDH1, two coactivators.^[^ ^13^ ^]^ The SAC monitors the attachment of spindle microtubules to chromosome kinetochores and does not initiate anaphase to prevent chromosome mis‐segregation until the spindle microtubules are accurately attached to the kinetochores. The SAC signal is inactivated when both kinetochores are correctly attached to the microtubules and when there is sufficient tension between the kinetochores. Under these conditions, the APC/C is activated by binding to CDC20 to form the APC/C^CDC20^ complex. The APC/C degrades Cyclin B1 and SECURIN, which in turn leads to the inactivation of CDK1 and the activation of Separase. Separase then cleaves the Kleisin subunit of cohesins, allowing the cell to proceed to anaphase.^[^ ^10^, ^14^ ^]^ In contrast to mitotic prometaphase where the primary APC species is APC^CDC20^,^[^ ^13^ ^]^ mammalian oocytes exhibit activity of APC^CDH1^ during prophase I and early prometaphase I prior to the involvement of APC^CDC20^.^[^ ^11^, ^14^, ^15^ ^]^ APC^CDH1^ targets CDC20 for degradation, but does not target SECURIN or Cyclin B1 in prophase I and early prometaphase I. The anaphase‐trigger is APC^CDC20^–mediated SECURIN or Cyclin B1 degradation, which is same as mitosis.^[^ ^11^, ^13^, ^15^ ^]^ Based on our findings and existing research, we inferred that the absence of NUSAP leads to downregulation of CDH1, which in turn leads to an increase in CDC20 and further acceleration of the degradation of SECURIN or Cyclin B1, thereby accelerating the meiotic progression of oocytes. Therefore, we further observed the protein levels of their regulatory protein CDH1 in NUSAP‐deficient oocytes. Immunoblotting showed that protein level of CDH1 was decreased in NUSAP‐deficient oocytes, and was restored in NUSAP‐rescue oocytes (Figure 5E). However, mRNA level of CDH1 was not affected by NUSAP deletion (Figure S7G, Supporting Information). This implies that NUSAP may be a new stabilizing factor for CDH1.

We next examined the expression patterns of CDC20, SECURIN, and Cyclin B1 in control and NUSAP‐deficient oocytes during oocyte meiosis. As shown in Figure 5F–H, 2–4 hours after GVBD, in control oocytes, CDC20 protein levels decreased significantly and SECURIN and Cyclin B1 increased obviously. At 4–8 hours after GVBD, CDC20 protein levels elevated, whereas SECURIN and Cyclin B1 protein levels increased first and decreased significantly after 6 hours of GVBD. In contrast, CDC20 persistently maintained high protein levels in NUSAP‐MO oocytes. SECURIN and Cyclin B1 maintained high levels at 2–4 hours of GVBD, but decreased significantly after 4 hours of GVBD. In NUSAP‐rescue oocytes, the immunoblots revealed a similar dynamic protein expression pattern for CDC20, Cyclin B1, and SECURIN as observed in the control group. There is still a question regarding whether the protein level of CDH1 is inversely correlated with that of CDC20 during the maturation process of oocytes. We conducted Western blot (WB) experiments at various stages of oocyte development and observed an interesting phenomenon: at 4 hours post‐GVBD, the protein level of CDC20 significantly decreased, but there was no significant change in the level of CDH1. When, at 6 hours post‐GVBD, the protein level of CDC20 significantly increased, the level of CDH1 significantly decreased at this time (Figure S9A, Supporting Information). Subsequently, we observed the protein levels of CDC20 by knocking down CDH1 in oocytes and found that in CDH1‐deficient oocytes, the protein levels of CDC20 were elevated (Figure S9B, Supporting Information). Overexpression of CDC20 in oocytes led to a decrease in the protein levels of Cyclin B1 and SECURIN, but had no effect on the protein levels of CDH1 (Figure S9C, Supporting Information). In summary, this suggests that a reduction in CDH1 protein levels can indeed result in an increase in CDC20 protein levels. It also suggests that the abundance of CDH1 protein is just one of the factors influencing the regulation of CDC20 protein levels, and that there may be other regulatory mechanisms at play, such as protein modification and dynamic changes in protein‐protein interactions, which affect protein abundance.

In conclusion, it is evident that the ultimate goal of the SAC is to prevent chromosome mis‐segregation and subsequent aneuploidy by regulating the timely degradation of cell cycle proteins such as Cyclin B1 and SECURIN. Although the deletion of NUSAP does not affect the localization of SAC proteins like BUBR1 to kinetochores, it ultimately leads to the premature degradation of Cyclin B1 and SECURIN by affecting the protein level of CDH1. This premature degradation accelerates the progression of oocytes, resulting in premature PBE, which in turn leads to aneuploidy. It can be seen that the deletion of NUSAP renders the SAC in name only. Thus, the absence of NUSAP leads to the loss of SAC's function in regulating the cell cycle, allowing oocytes to evade its monitoring.

To investigate whether NUSAP binds to CDH1, and to further clarify whether NUSAP can directly bind to SECURIN and Cyclin B1 and regulate their protein abundance independently of CDH1. We performed yeast two‐hybrid (Y2H) experiments of NUSAP with CDH1, SECURIN and Cyclin B1. NUSAP was fused to Gal4‐BD as the bait protein. CDH1, SECURIN or Cyclin B1 were separately fused to Gal4‐AD as the prey protein. As shown in Figure 5I, NUSAP can interact with CDH1 but not with SECURIN and Cyclin B1. Subsequently by microinjection to exogenously express NUSAP and CDH1 and performing co‐immunoprecipitation (IP) experiments, we further confirmed the interaction of NUSAP with CDH1 in oocytes (Figure 5J).

### 2.6 Elevation of CDH1 Protein Level Rescues NUSAP Depletion‐Induced Meiotic Defects and Aneuploidy

**Figure 6:**
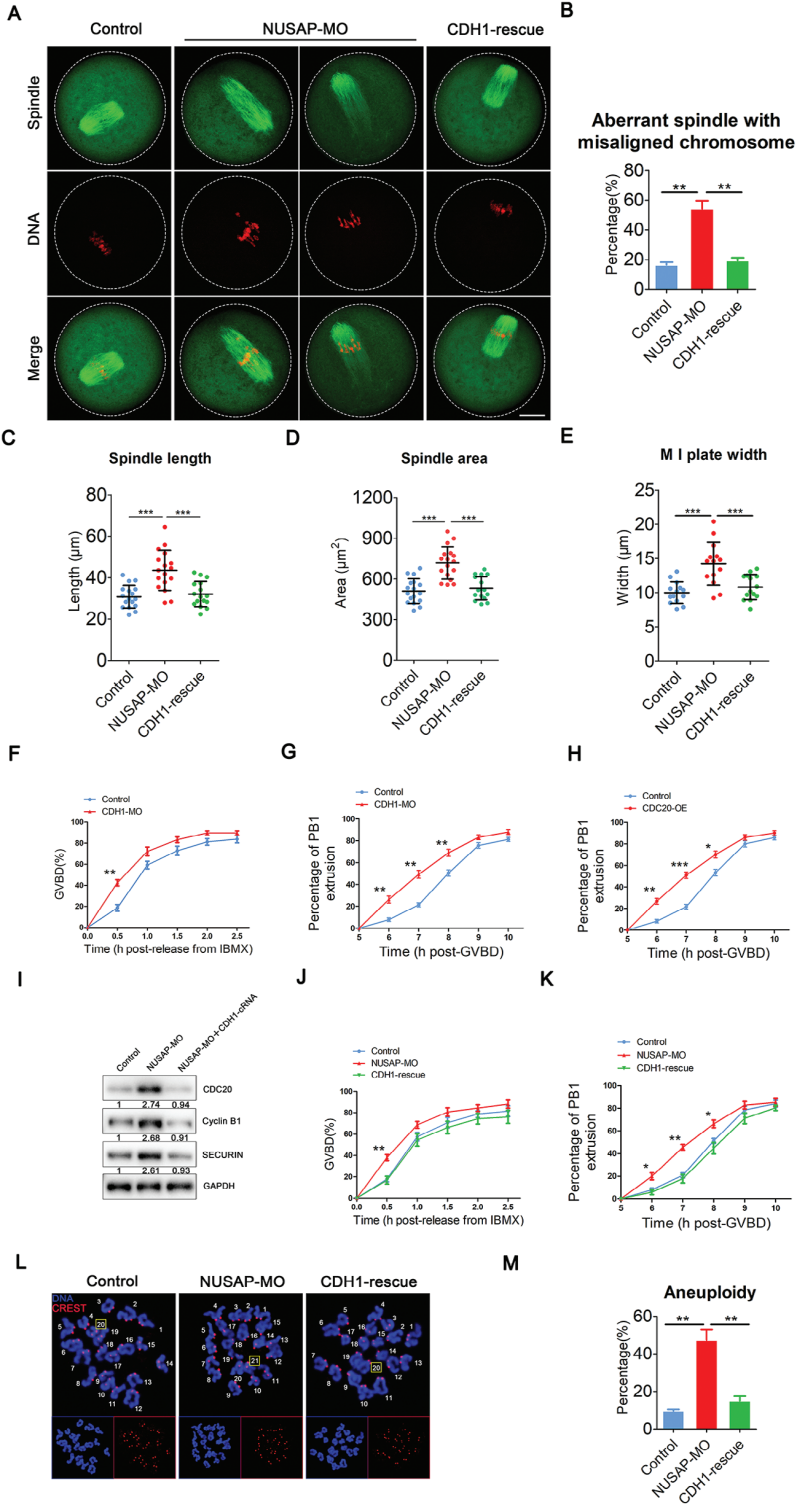
Accelerated meiotic progression and aneuploidy induced by NUSAP depletion could be restored by expression of exogenous CDH1. A) Representative images of spindle morphologies and chromosome alignment in control, NUSAP‐MO, and CDH1‐rescue oocytes. At 6 hours post‐GVBD, oocytes were fixed and immunostained for α‐tubulin and DNA (PI). Scale bar, 20 µm. B) The rates of aberrant spindle with misaligned chromosome were recorded in control (n = 61), NUSAP‐MO (n = 59), and CDH1‐rescue (n = 52) oocytes. C,D) The spindle length and area were measured in control (n = 17), NUSAP‐MO (n = 17) and CDH1‐rescue (n = 16) oocytes at 6 hours post‐GVBD. E) The width of M I plate was measured in control (n = 14), NUSAP‐MO (n = 14), and CDH1‐rescue (n = 14) oocytes. F) The incidence of GVBD at 0.5, 1, 1.5, 2 and 2.5 hours post‐IBMX release was quantified in control (n = 118) and CDH1‐MO (n = 108) oocytes. G) Quantitative analysis of PBE rate was shown in control (n = 127) and CDH1‐MO (n = 122) oocytes at consecutive time points of post‐GVBD. H) Quantitative analysis of PBE rate was shown in control (n = 120) and CDC20‐OE (n = 120) oocytes at consecutive time points of post‐GVBD. I) Protein levels of CDC20, Cyclin B1and SECURIN in control, NUSAP‐MO and NUSAP‐MO +CDH1‐cRNA oocytes at GV stage. The blots were probed with CDC20, Cyclin B1, SECURIN, and GAPDH antibodies. J) The incidence of GVBD at 0.5, 1, 1.5, 2, and 2.5 hours post‐IBMX release was quantified in control (n = 122), NUSAP‐MO (n = 108) and CDH1‐rescue (n = 120) oocytes. For the rescue experiment, GV oocytes were injected with NUSAP‐specific morpholino oligonucleotides and maintained for 20 hours in 50 µm IBMX before being injected with CDH1 cRNA and maintained for a further 3 hours in 200 µm IBMX to allow time for translation. Oocytes were then washed into IBMX‐free medium to allow resumption of meiosis. K) Quantitative analysis of PBE rates were shown in control (n = 125), NUSAP‐MO (n = 121) and CDH1‐rescue (n = 118) oocytes at consecutive time points of post‐GVBD. L) Representative images of euploid and aneuploid M II eggs. Chromosome spreading was performed to count the number of chromosomes in control, NUSAP‐MO and CDH1‐rescue oocytes at 10 hours after GVBD. The total number of univalents is indicated by the yellow square. Scale bar, 5 µm. M) The rates of aneuploid eggs were recorded in control (n = 31), NUSAP‐MO (n = 30) and CDH1‐rescue (n = 26) oocytes. Data were presented as mean percentage (mean ± SEM) of at least three independent experiments. **p < 0.01.

The above experiments imply that the meiotic defects caused by NUSAP depletion may involve the downregulation of CDH1 protein levels. If decreased protein levels of CDH1 caused by depletion of NUSAP cause meiotic defects and aneuploidy, then introduction of exogenous CDH1 into NUSAP‐depleted oocytes should reverse the phenotype. In order to verify this assumption, we examined meiotic progression after microinjection of CDH1 mRNA into the NUSAP‐depleted oocytes (CDH1‐rescue oocytes). The higher frequency of abnormal spindles and misaligned chromosomes observed in NUSAP‐depleted oocytes was reduced to a level indistinguishable from controls in CDH1‐rescue oocytes (**Figure** **6**A,B). In addition, the defective spindle length, spindle area, and width of chromosome plate at M I stage was restored in CDH1‐rescue oocytes (Figure 6C–E). Moreover, CDH1 knockdown oocytes exhibit abnormal spindle assembly and chromosome alignment similar to that observed in NUSAP knockdown (Figure S10, Supporting Information). As shown in Figure 6F,G, CDH1 knockdown also resulted in accelerated GVBD and PBE, which has a similar phenotype to NUSAP depletion. Due to reduced protein levels of CDH1 in oocytes lead to increased CDC20 protein levels (Figure S9B, Supporting Information), and we next evaluated the effect of CDC20 overexpression on PBE and found that CDC20 overexpression also caused premature PBE (Figure 6H), as well as a significant increase in aneuploid oocytes (Figure S9D, Supporting Information).

We further observed whether exogenous expression of CDH1 in NUSAP‐deficient oocytes could remedy the imbalance in abundance of cell cycle proteins caused by NUSAP depletion. According to immunoblotting data, NUSAP‐deficient oocytes with exogenous CDH1 expression restored CDC20, SECURIN, and Cyclin B1 to levels that were comparable to those of control oocytes (Figure 6I). As shown in Figure 6J, oocytes rescued by CDH1 restored the accelerated GVBD in NUSAP‐depleted oocytes to the level of control oocytes, implying that the accelerated GVBD caused by the absence of NUSAP involves the APC^CDH1^ regulation.

Furthermore, the accelerated PBE at 6 to 8 hours after GVBD observed in NUSAP‐depleted oocytes recovered in CDH1‐rescue oocytes to that of control oocytes (Figure 6K). Ultimately, expression of exogenous CDH1 rescued the high incidence of aneuploidy exhibited in NUSAP‐depleted oocytes to comparable levels of control oocytes (Figure 6L,M).

In summary, the above experimental results indicate that restoring CDH1 protein levels can rescue the defects caused by NUSAP depletion in oocytes, suggesting that NUSAP's role in preventing oocytes from evading the SAC and controlling the meiotic process is mediated by CDH1.

### 2.7 NUSAP Protects CDH1 from SCF^BTRC^‐Mediated Degradation

**Figure 7:**
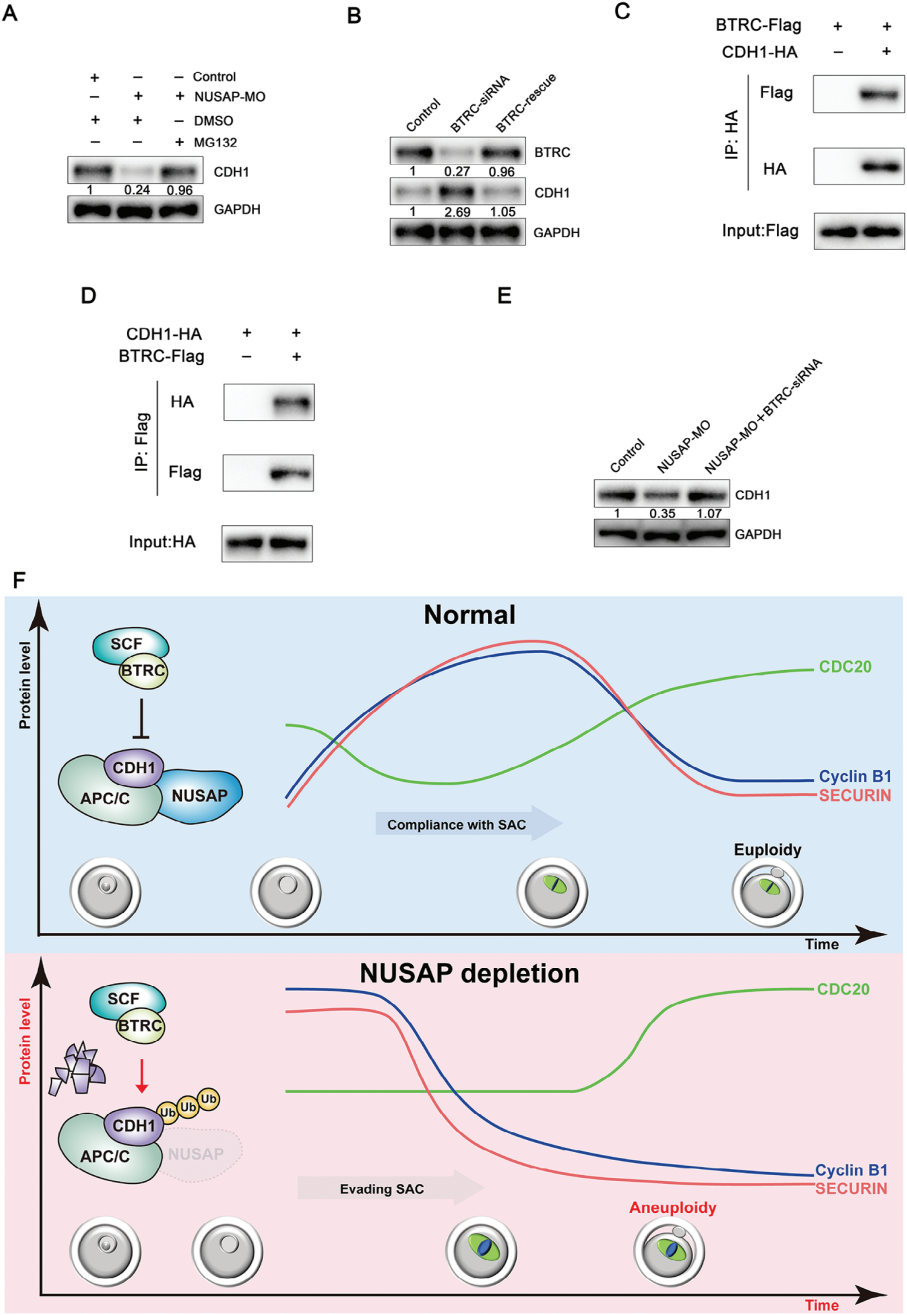
NUSAP protects CDH1 from SCF^BTRC^‐mediated degradation. A) Protein levels of CDH1 in control, NUSAP‐MO, and NUSAP‐MO + MG132 oocytes at GV stage. GV oocytes were injected with control or NUSAP‐specific morpholinos, maintained for 20 h in 50 µm IBMX and 25 µm MG132 (S2619, Selleck). The blots were probed with CDH1 and GAPDH antibodies. B) Protein levels of CDH1 in control, BTRC‐siRNA, and BTRC‐rescue oocytes at GV stage. The blots were probed with BTRC, CDH1, and GAPDH antibodies. C) Co‐immunoprecipitation result showing CDH1 interaction with BTRC in oocytes. Mouse oocytes were microinjected with CDH1‐HA and BTRC‐Flag cRNA together or CDH1‐HA cRNA alone, maintained for a further 4 hours in 200µm IBMX to allow time for translation. Target proteins were immunoprecipitated using anti‐HA beads and subjected to western blotting with HA and Flag antibodies. Input oocyte lysates were immunoblotted with anti‐Flag antibody to determine the expression of BTRC. D) Co‐immunoprecipitation result showing BTRC interaction with CDH1 in oocytes. Mouse oocytes were microinjected with BTRC‐Flag and CDH1‐HA cRNA together or BTRC‐Flag cRNA alone, maintained for a further 4 hours in 200µm IBMX to allow time for translation. Target proteins were immunoprecipitated using anti‐Flag beads and subjected to western blotting with Flag and HA antibodies. Input oocyte lysates were immunoblotted with anti‐ HA antibody to determine the expression of CDH1. E) Protein levels of CDH1 in control, NUSAP‐MO, and NUSAP‐MO + BTRC‐siRNA oocytes. The blots were probed with CDH1 and GAPDH antibodies. F) Working model of the mechanism that microtubule‐associated protein NUSAP governs proper cell cycle speed to ensure oocyte euploidy. In normal oocytes, NUSAP interacts with CDH1 and protects CDH1 from SCF^BTRC^‐mediated degradation to regulate APC/C activity, normal spindle assembly and chromosome alignment and the appropriate abundance of cell cycle proteins for proper cell cycle speed to ensure oocyte euploidy. However, depletion of NUSAP results in aneuploid eggs due to SCF^BTRC^‐mediated degradation of CDH1, subsequent abnormal spindle assembly and chromosome alignment and abnormal cell cycle protein abundance. The deletion of NUSAP does not affect the localization of SAC proteins to kinetochores. However, due to the abnormal cell cycle protein abundance, the oocytes evade the surveillance of the Spindle Assembly Checkpoint (SAC), rendering the SAC in name only. This leads to oocytes with abnormal spindles and misaligned chromosomes not only failing to be arrested at metaphase, but also accelerating the meiotic process of the oocytes and ultimately resulting in the production of aneuploid eggs.

After NUSAP depletion, CDH1 mRNA levels remained unchanged (Figure S7G, Supporting Information), but ubiquitination and subsequent degradation were perturbed. Treatment with the proteasome inhibitor MG132 significantly elevated CDH1 protein levels in NUSAP‐depleted oocytes, as shown by immunoblotting (**Figure** **7**A). CDH1 is a known substrate of the E3 ubiquitin ligase SCF^BTRC^ in mitosis.^[^ ^16^ ^]^ To explore whether NUSAP can protect CDH1 from SCF^BTRC^‐mediated degradation in oocytes, we then used RNAi to deplete BTRC. Increased protein levels of CDH1 were observed in BTRC‐depleted oocytes, and protein levels of CDH1 were close to normal in BTRC‐rescue oocytes (Figure 7B). Furthermore, co‐immunoprecipitation results demonstrated an interaction between CDH1 and BTRC in oocytes (Figure 7C). Reciprocal experiments confirmed their interaction (Figure 7D). The protein levels of CDH1 were restored to levels comparable to those in control oocytes in NUSAP‐MO + BTRC‐siRNA oocytes (Figure 7E).

## 3 Discussion

Meiosis involves a single round of DNA replication followed by two sequential nuclear and cellular divisions, known as meiosis I and meiosis II.^[^ ^10^, ^17^ ^]^ Oocyte meiosis I is characterized by first meiosis resumption and segregation of homologous chromosomes (bivalents), followed by meiosis II arrest at metaphase when sister chromatids remain united. Accurate bivalent segregation requires correct kinetochore microtubule attachments.^[^ ^10^, ^12^ ^]^ Incorrect or unstable K‐MT attachments contribute to bivalent misalignment and discordant divisions, which cause aneuploidy in mammalian eggs.^[^ ^10a,b^ ^]^ Understanding the mechanisms underlying this unique chromosome segregation is crucial for comprehending germ cell development.^[^ ^18^ ^]^

Microtubule‐associated proteins have been recognized for their ability to bind to microtubules, providing them with increased stability. These proteins are considered to be multifunctional organizers of the microtubule cytoskeleton, playing a crucial role in a variety of cellular activities, including spindle assembly, intracellular transport, neuronal development, and the formation of ciliary axonemes.^[^ ^1b,c^ ^]^ Unexpectedly, we revealed previously unknown biological functions of microtubule‐associated proteins, namely, that the microtubule‐associated protein NUSAP can prevent oocytes from evading the SAC and maintaining proper cell cycle speed. The NUSAP protein, known as a highly conserved microtubule‐associated protein in vertebrates, plays a crucial role in organizing and connecting microtubules to chromosomes.^[^ ^2^ ^]^

We investigated the subcellular distribution, expression, and function of NUSAP during female meiosis I in mice. The localization of NUSAP in oocytes has many similarities with that in somatic cells, its expression level significantly increases after GVBD, and it exhibits functions that are very different from those in somatic cells. The depletion of NUSAP resulted in abnormal spindle assembly and chromosome alignment as well as the acceleration of cell cycle progression. This finding is quite intriguing, as there have been no prior reports of microtubule‐binding proteins exerting repressive effects on the progression of the cell cycle.

Accelerated cell cycle is often associated with SAC dysfunction. SAC is a guarding machine that senses the correct attachment of spindle microtubules to kinetochores, thus licensing the cells to progress into anaphase and ensuring the accurate chromosome segregation. Dysregulation of SAC often leads to aneuploidy, while irregularities during mitosis potentially contribute to cell cancerization. Disruptions occurring during the process of oocyte meiosis may lead to trisomy syndrome or miscarriage.^[^ ^14^, ^19^ ^]^ Premature PBE in oocytes is highly correlated with the SAC dysfunction.^[^ ^12^, ^20^ ^]^

To clarify whether NUSAP depletion‐caused meiosis acceleration and aneuploidy is due to the dysfunction of SAC, we subsequently examined the protein levels of BUBR1, BUB3, MAD2 and CDC20, the components of mitotic checkpoint complex (MCC) in NUSAP‐deficient oocytes. Their protein levels were all increased (Figure 5C). Furthermore, the recruitment of several SAC proteins to unattached kinetochores during prometaphase I was observed in both control and NUSAP‐deficient oocytes (Figure 5C; Figure S8, Supporting Information), indicating that the absence of NUSAP did not impact their kinetochore recruitment. This is an unexpected phenomenon, which means that the absence of NUSAP does not directly lead to the inactivation of the SAC. So why does NUSAP cause the acceleration of the meiotic process in oocytes?

The progression of the cell cycle is facilitated by a protein degradation system known as the APC/C, which collaborates with two coactivators, CDC20 or CDH1.^[^ ^13^ ^]^ In contrast to mitotic prometaphase where APC^CDC20^ is the primary APC species,^[^ ^13^ ^]^ mammalian oocytes exhibit APC^CDH1^ activity during prophase I and early prometaphase I before APC^CDC20^ comes into play.^[^ ^11^, ^14^, ^15^ ^]^ During this stage, APC^CDH1^ specifically targets CDC20 for degradation while leaving SECURIN or Cyclin B1 untouched. The initiation of anaphase relies on APC^CDC20^‐mediated degradation of SECURIN or Cyclin B1, similar to what occurs in mitosis. When confined within the MCC, CDC20 encounters a hindrance in its co‐activation of APC/C. The APC/C loses its ability to designate various substances for degradation when CDC20 is absent, thereby impeding progress into anaphase. Specifically, the APC/C^CDC20^ alliance disintegrates SECURIN or Cyclin B1, which are required for anaphase onset.^[^ ^11^, ^13^, ^15^ ^]^ Based on the above, we deduced that the lack of NUSAP results in the reduction of CDH1, consequently causing an elevation in CDC20 and facilitating the degradation process of SECURIN or Cyclin B1. As a result, this expedites the advancement of oocyte meiotic progression. A series of tests and rescue experiments did confirm our hypothesis. Although the deletion of NUSAP does not affect the localization of SAC proteins to kinetochores, it leads to the premature degradation of Cyclin B1 and SECURIN by affecting the protein level of CDH1. This premature degradation accelerates the progression of oocytes, resulting in premature PBE. It can be seen that the deletion of NUSAP renders the SAC in name only, causing oocytes to evade SAC surveillance. This results in oocytes with abnormal spindles and misaligned chromosomes not only failing to be arrested at metaphase, but also accelerating the meiotic speed and ultimately resulting in the production of aneuploid eggs.

Last, we elucidate the specific mechanism by which NUSAP maintains the stability of CDH1. NUSAP can effectively protect CDH1 from degradation mediated by the E3 ubiquitin ligase SCF^BTRC^. The E3 ubiquitin ligase complex SCF and APC/C are two very important regulators responsible for the control of the cell cycle.^[^ ^21^ ^]^ Investigating their harmonious regulation is key to understanding how the cell correctly progresses through the cell cycle, and the role of NUSAP in protecting CDH1 from degradation by the E3 ubiquitin ligase SCF^BTRC^ indicates that it is an important regulatory factor in coordinating these two major cell cycle regulators. NUSAP deficiency in mice causes early embryonic lethality, ongoing spindle checkpoint activity and mitotic arrest, which ultimately triggers caspase induction and cell death through apoptosis.^[^ ^5^ ^]^ This implies that NUSAP does not function to prevent somatic cells from evading the surveillance of the SAC; otherwise, mitosis would proceed to anaphase. NUSAP plays a novel role in oocytes, serving as an additional ‘safety’ measure beyond SAC surveillance, which can significantly reduce the likelihood of oocytes escaping SAC and producing aneuploidy. Previous studies have shown that NUSAP is a substrate of APC/C in mitosis and its protein level is precisely regulated by APC/C.^[^ ^22^ ^]^ Unlike in somatic cells, in oocytes, NUSAP's maintenance of CDH1 protein homeostasis influences APC/C, which is crucial for NUSAP's role in preventing oocytes from escaping SAC. Compared to somatic cells, oocytes are unique, characterized by a larger volume, less precise SAC activity,^[^ ^14a^ ^]^ and a longer division phase. Human oocytes, compared to those of other mammals, have a higher rate of aneuploidy, and the human oocyte's more unstable spindle and unique spindle assembly form may be significant factors,^[^ ^23^ ^]^ although other factors cannot be ruled out, such as the lower NUSAP protein content in human oocytes compared to those of other mammals. Exploring the specific causes of these differences is an interesting research topic.

In summary, we reveal that NUSAP, a microtubule‐binding protein, orchestrates SAC and the speed of the cell cycle by maintaining CDH1 levels in oocytes. Loss of NUSAP results in the production of aneuploid eggs due to SCF^BTRC^‐mediated degradation of CDH1 (Figure 7F). Our findings provide novel insights into the noncanonical role of NUSAP in preventing oocytes from evading the SAC and maintaining proper cell cycle speed, thereby ensuring the production of euploid oocytes.

## 4 Experimental Section

### Animals

6 to 8‐week‐old female ICR mice were used in all experiments, which were approved by the Animal Care and Use Committee of Guangdong Second Provincial General Hospital and performed in accordance with institutional guidelines.

### Oocyte Collection and Culture

Female ICR mice were sacrificed by cervical dislocation. Fully‐grown oocytes arrested at prophase of meiosis I were collected from ovaries in M2 medium (Sigma‐Aldrich, St. Louis, MO, USA). Only those immature oocytes displaying a germinal vesicle (GV) were further cultured in M16 medium (Sigma‐Aldrich) under liquid mineral oil at 37 °C in an atmosphere of 5% CO_2_. At different time points after culture, oocytes were collected for subsequent analysis.

### Morpholino Knockdown

NUSAP‐targeting morpholino antisense oligo (Gene Tools, Philomath, OR, USA; 5’‐ CGTTCTGCAATCTCGGTGATTCCCA ‐3’) or CDH1‐targeting morpholino antisense oligo (Gene Tools, Philomath, OR, USA; 5’‐ CCTTCGCTCATAGTCCTGGTCCATG ‐3’) was diluted with water to give a working concentration of 1 mM, and then approximately 5–10 pl of oligo was microinjected into the cytoplasm of fully grown GV oocytes using a Narishige microinjector (Tokyo, Japan). A non‐targeting morpholino oligo (5’‐CCTCTTACCTCAGTTACAATTTATA‐3’) was injected as a control. In order to facilitate the morpholino‐mediated inhibition of mRNA translation, oocytes were arrested at GV stage in M16 medium containing 50 µM IBMX for 20 hours, and then cultured in IBMX‐free M16 medium for subsequent experiments.

### siRNA Knockdown

NUSAP‐targeting siRNA antisense oligo (Genepharma, Shanghai, China; 5’‐GCACACCUGAAUCCAGAAATT‐3’; 5’‐GGAGAUGAAAGGAACUGAUTT‐3’) was diluted with water to give a working concentration of 25 µM, and then approximately 5–10 pl of oligo was microinjected into the cytoplasm of fully grown GV oocytes using a Narishige microinjector (Tokyo, Japan). A non‐targeting siRNA oligo (antisense sequence: 5’‐ACGUGACACGUUCGGAGAATT‐3’) was injected as a control. In order to facilitate the degradation of mRNA by siRNA, oocytes were arrested at GV stage in M16 medium containing 50 µM IBMX for 20 hours, and then transferred to IBMX‐free M16 medium to resume the meiosis for subsequent experiments.

### cRNA Construct and In Vitro Transcription

cDNA was subcloned into pcDNA3.1. Mutant NUSAP with four silent third‐codon point mutations in the sequence targeted by the morpholino provided a MO‐resistant construct. Capped cRNA was synthesized from linearized plasmid using T7 mMessage mMachine kit (ThermoFisher Scientific, Waltham, MA, USA), and purifed with MEGAclear kit (ThermoFisher Scientific). Typically, 10–12 pl of 0.5‐1.0 µg/µl cRNA was injected into oocytes and then arrested at GV stage in M16 medium containing 200 µM IBMX for 2–4 hours to allow translation prior to transfer into IBMX‐free M16 medium for subsequent studies.

### Immunofluorescence and Confocal Microscopy

Oocytes were fixed in 4% paraformaldehyde in PBS (pH 7.4) for 30 min and permeabilized in 0.5% Triton‐X‐100 for 20 min at room temperature. Then, oocytes were blocked with 1% BSA‐supplemented PBS for 1 hour and incubated with NUSAP (1:100; ProteinTech, 12024‐1‐AP), BUBR1 (1:100; Abcam, Ab28193), MAD2 (1:50; ProteinTech, 10337‐1‐AP), BUB1 (1:100; Abcam, ab195268), MPS1 (1:50; ProteinTech, 10381‐1‐AP), α‐tubulin‐FITC (1:300; Sigma‐Aldrich, F2168) or CREST (1:200; Antibodies Incorporated, CA95617), antibodies at 4 °C overnight. After washing in PBST, oocytes were incubated with an appropriate secondary antibody for 1 hour at room temperature. Then oocytes were counterstained with PI or Hoechst for 10 min. Finally, oocytes were mounted on glass slides and observed under a confocal microscope (LSM 900 META, Zeiss, Germany).

For measurement of fluorescence intensity, the signals from both control and treatment oocytes were acquired by performing the same immunostaining procedure and setting up the same parameters of confocal microscope. The average fluorescence intensity per unit area within the region of interest (ROI) was applied to quantify the fluorescence of each oocyte images. Fluorescence intensity was randomly measured by plot profiling using ImageJ software (NIH, USA). Fluorescence intensity on kinetochores was quantified by drawing a circle closely the dot‐like CREST staining covering the interested SAC proteins staining. The intensity of SAC proteins was normalized against the CREST fluorescence intensity.

### Yeast Two‐Hybrid Assay

The NUSAP CDS was cloned into the pGBKT7 vector, while the CDH1, Cyclin B1, and SECURIN CDSs were cloned into the pGADT7 vector. The resulting plasmids were co‐transformed in the appropriate pairs into yeast strain Y2HGold, followed by selection on synthetic defined medium lacking leucine and tryptophan (SD/‐Leu/‐Trp). Positive colonies were spotted onto SD/‐Leu/‐Trp medium and SD/‐Ade/‐His/‐Leu/‐Trp medium and grown for 3–4 days at 30°C. The pGBKT7‐p53 and pGADT7‐LargeT clones were used as the positive control, while pGBKT7‐LaminC and pGADT7‐LargeT were used as the negative control. The pGBKT7‐NUSAP and pGADT7 clones were used to test whether pGBKT7‐NUSAP was self‐activated.

### Immunoprecipitation and Immunoblotting Analysis

For co‐immunoprecipitation, 300 mouse oocytes were microinjected with NUSAP‐Flag and CDH1‐HA cRNA together or CDH1‐HA cRNA alone, maintained for a further 4 hours in 200µM IBMX to allow time for translation. Then those oocytes were harvested in lysis buffer containing a protease inhibitor cocktail (Invitrogen). Target proteins were immunoprecipitated using anti‐Flag beads were incubated together for 16 hours at 4 °C. After five washes with wash buffer, the bead‐antibody‐antigen complex were then resuspended in elution buffer. Samples were supplemented with 4 × LDS sample buffer (ThermoFisher) and heated at 95 °C for 5min. For immunoblots, samples were separated on 10% Bis‐Tris precast gels and transferred onto PVDF membranes. The blots were further blocked in TBST containing 5% low fat dry milk for 1 hour at room temperature and then incubated with NUSAP (1:500; ProteinTech, 12024‐1‐AP), CDH1 (1:1000; Abcam, ab77885), Cyclin B2 (1:1000; Abcam, ab185622), Cyclin B1 (1:1000; Cell Signaling Technology, 4135), BUBR1 (1:1000; Abcam, Ab28193), BUB3 (1:1000; Abcam, ab133699) MAD2 (1:1000; ProteinTech, 10337‐1‐AP), SECURIN (1:1000; Abcam, ab79546), pCDK1 (1:1000; Cell Signaling Technology, 9111), CDC20 (1:500; ProteinTech, 10252‐1‐AP), Cofilin (1:5000; ProteinTech, 66057‐1‐Ig), BTRC (1:1000; Cell Signaling Technology, 4394) or GAPDH (1:5000; Cell Signaling Technology, 2118) antibodies at 4 °C overnight. After washing in TBST, the blots were incubated with HRP conjugated secondary antibodies for 1 hour at room temperature. Chemiluminescence was detected with ECL Plus (GE, Piscataway, NJ, USA) and protein bands were acquired by Tanon‐3900 Chemiluminescence Imaging System (Tanon, Beijing, China). Band intensities were quantified using ImageJ software and normalized to loading controls. For the immunoblotting experiments, they were repeated twice, and the results were consistent.

### RT‐qPCR

10–20 oocytes collected were lysed in cell lysis buffer containing 0.2% Triton X‐100 and RNase inhibitor. The lysate was then reverse transcribed using SuperScript III First‐Strand System (Invitrogen, 18080051) following manufacturers’ instructions. Quantitative PCR was conducted with One Step TBGreen PrimeScript RT‐PCR Kit (Takara, RR066A) on ABI QuantStudio5 Real‐Time PCR system (Applied Biosystems). Murine Atcb was applied as an endogenous control. Data were obtained from at least three replicated biological experiments per genotype with three technical repeats each time and were expressed as enrichment of 2^−ΔΔCt^.

### Chromosome Spreading

Oocytes were exposed to Tyrode's buffer (pH 2.5) for about 30 seconds at 37 °C to remove zona pellucidae. After recovery in M2 medium for 10 min, oocytes were fixed in a drop of 1% paraformaldehyde with 0.15% Triton X‐100 on a glass slide. After air drying, chromosomes were counterstained with Hoechst and examined under a laser scanning confocal microscope.

### Statistical Analysis

All percentages or values from at least three biological replicates were expressed as mean ± SEM or mean ± SD, and the number of oocytes was labeled in parentheses as (n). Data were analyzed by paired‐samples t‐test, which was provided by GraphPad Prism 5 statistical software. The level of significance was accepted as *p* < 0.05.

## Conflict of Interest

The authors declare no conflict of interest.

## Author Contributions

C.Z., X.Z., G.X., Y.R., and H.W. contributed equally to this work. C.Z. and Q.S. conceived and designed the project. C.Z. wrote the manuscript and Q.S. reviewed and edited the manuscript. C.Z., X.Z., Y.R., and H.W. performed most of the experiments. C.Z., Q.S., and X.O. supervised this project. All authors discussed the results and contributed to the final manuscript.

## Supporting information



Supporting Information

## Data Availability

The data that support the findings of this study are available in the supplementary material of this article.
