# Supplementary material for: A Microtubule‐Associated Protein Functions in Preventing Oocytes from Evading the Spindle Assembly Checkpoint
Source: Adv Sci (Weinh). 2024 Dec 25;12(7):2413097. doi: 10.1002/advs.202413097 (PMC11831433; doi:10.1002/advs.202413097)
Supplement: Supplementary file 1 — Supporting Information [file ADVS-12-2413097-s001.docx]

**Supporting Information for**

A microtubule- associated protein functions in preventing oocytes from evading the spindle assembly checkpoint

Changyin Zhou*****, Xue Zhang, Genlu Xu, Yuting Ran, Hui Wang, Xuefeng Xie, Ang Li, Fei Li, Xiaozhen Li, Jinlong Ding, Mianqun Zhang, Qing-Yuan Sun*****, Xiang-Hong Ou*****

***** Changyin Zhou

**Email:** changyinzhou@126.com

*****Xiang-Hong Ou

**Email:** ouxh@gd2h.org.cn

*****Qing-Yuan Sun

**Email:** sunqy@gd2h.org.cn

**This PDF file includes:**

Figures S1 to S10


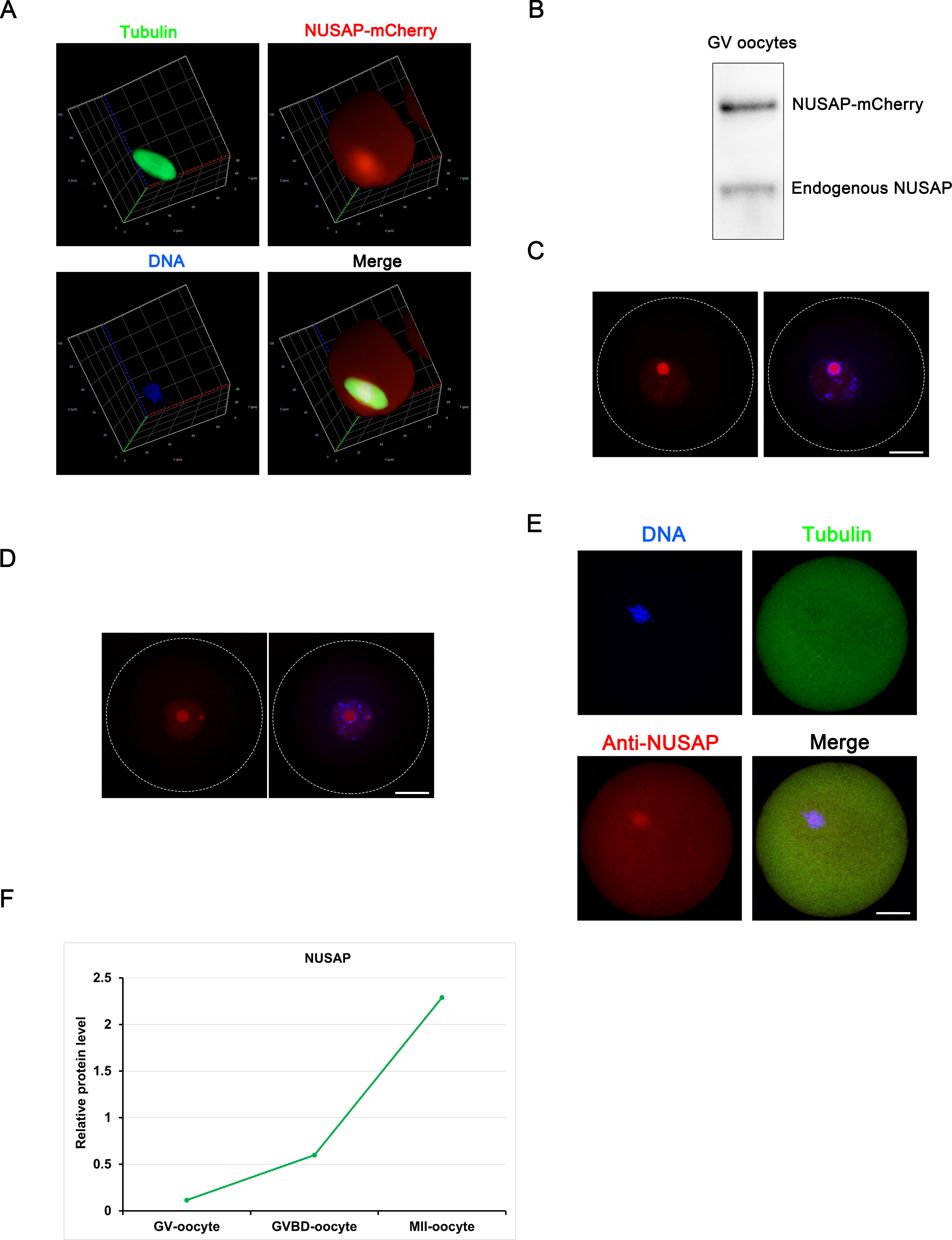


**Figure S1. Localization and expression of NUSAP during oocyte meiosis.** (A) Representative 3D modeling images of NUSAP-mCherry localization in mouse oocyte at 6 hours after GVBD. (B) GV stage oocytes injected with low concentrations of NUSAP-mcherry cRNA were incubated with anti-NUSAP antibody. (C) Representative images show the subcellular localization of NUSAP-mCherry at the GV stage, when the expression level of NUSAP-mCherry is close to that of the endogenous NUSAP protein. Scale bar, 20 μm. (D) Representative images show the subcellular localization of NUSAP-mCherry in NUSAP-MO oocyte at GV stage. Scale bar, 20 μm. (E) Representative images of NUSAP localization by nocodazole treatment in oocytes. Scale bar, 20 μm. (F)The Relative protein level of NUSAP in Proteomics data at each developmental stage of mouse oocytes.


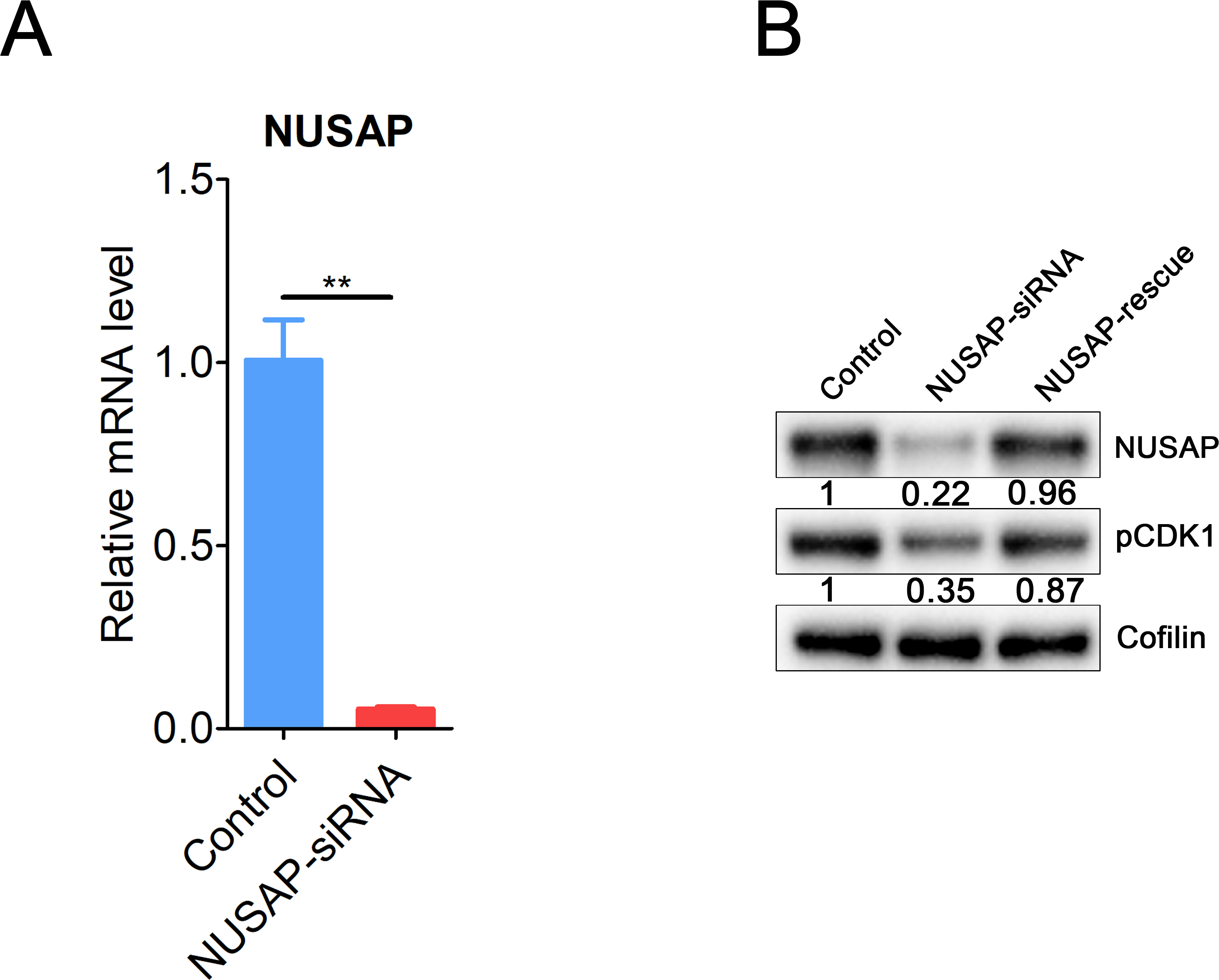


**Figure S2. Knockdown efficiency of NUSAP-targeting siRNA in mouse oocytes.** (A) Reverse transcription polymerase chain reaction (RT-PCR) showing the Relative mRNA level of NUSAP in control and NUSAP-siRNA groups. (B) Oocytes in control, NUSAP-siRNA and NUSAP-rescue groups were collected and immunoblotted for NUSAP, pCDK1 and Cofilin. Data were presented as mean percentage (mean ± SEM) of at least three independent biological replicates. ***P* < 0.01.


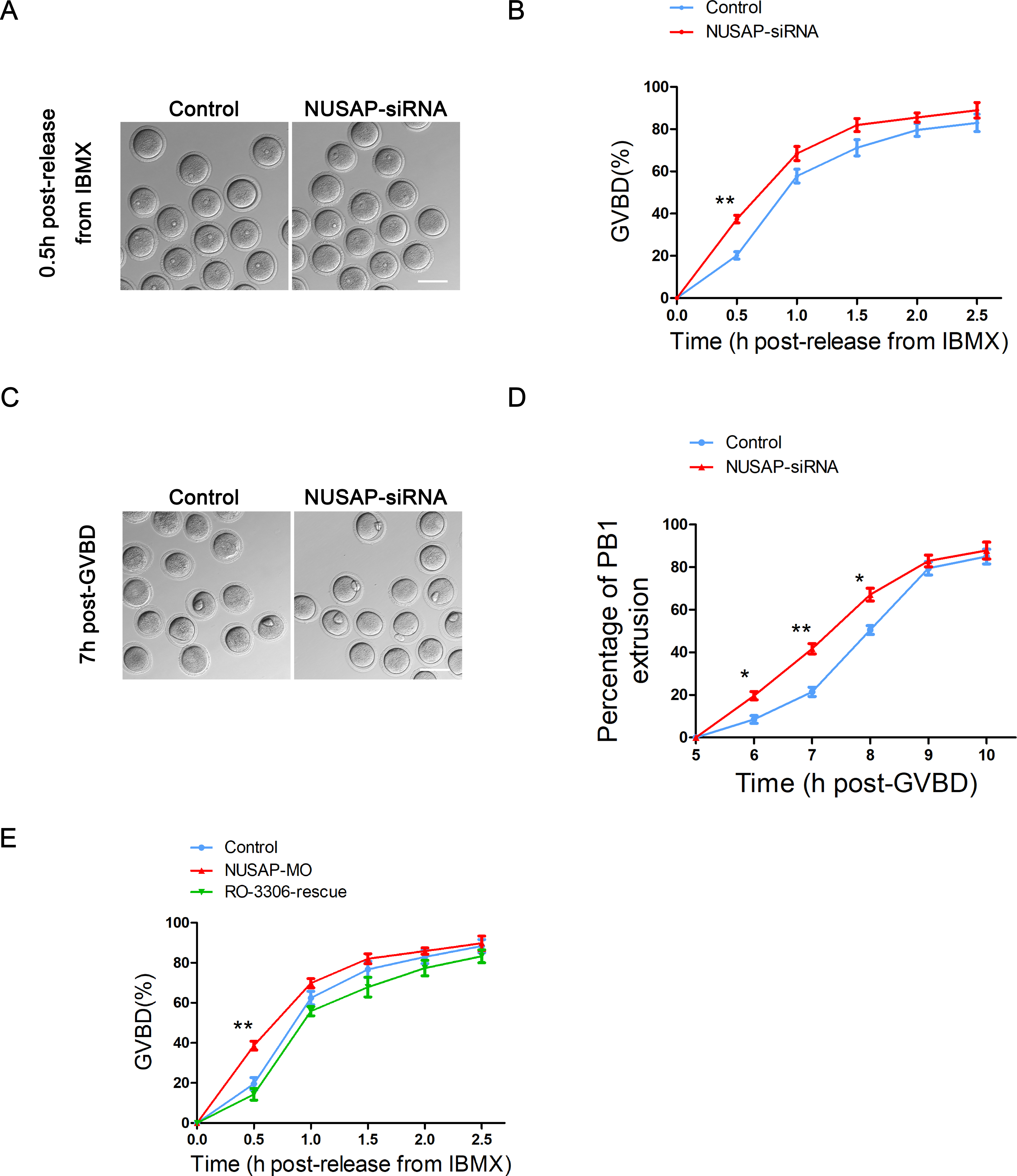


**Figure S3. Effect of NUSAP depletion on meiotic progression in mouse oocytes.** (A) Representative images of the occurrence of GVBD in control and NUSAP-siRNA oocytes at 0.5 h following release from IBMX. Scale bar, 100 μm. (B) The incidences of GVBD at 0.5, 1, 1.5, 2 and 2.5 hours post-IBMX release were quantified in control (n = 119) and NUSAP-siRNA (n = 112) oocytes. (C) Representative images of PBE in control and NUSAP-siRNA oocytes at the time point of 7 h post-GVBD. Scale bar, 100 μm. (D) Quantitative analysis of PBE rates was shown in control (n = 107) and NUSAP-siRNA (n = 101) oocytes at consecutive time points of post-GVBD. (E) The incidence of GVBD at 0.5, 1, 1.5, 2 and 2.5 hours post-IBMX release was quantified in control (n = 112), NUSAP-MO (n = 106) and RO-3306-rescue (n = 116) oocytes. For the rescue experiment, GV oocytes were injected with NUSAP-specific morpholino oligonucleotides and maintained in 50 µM IBMX for 20 hours. They were then treated with 100 nM RO-3306 (S7747; Selleck) and 50 µM IBMX for 1 hour at the GV stage. After this treatment, the oocytes were washed into a medium containing 100 nM RO-3306 but without IBMX to allow resumption of meiosis. Data were presented as mean percentage (mean ± SEM) of at least three independent biological replicates. **P* < 0.05, ***P* < 0.01.


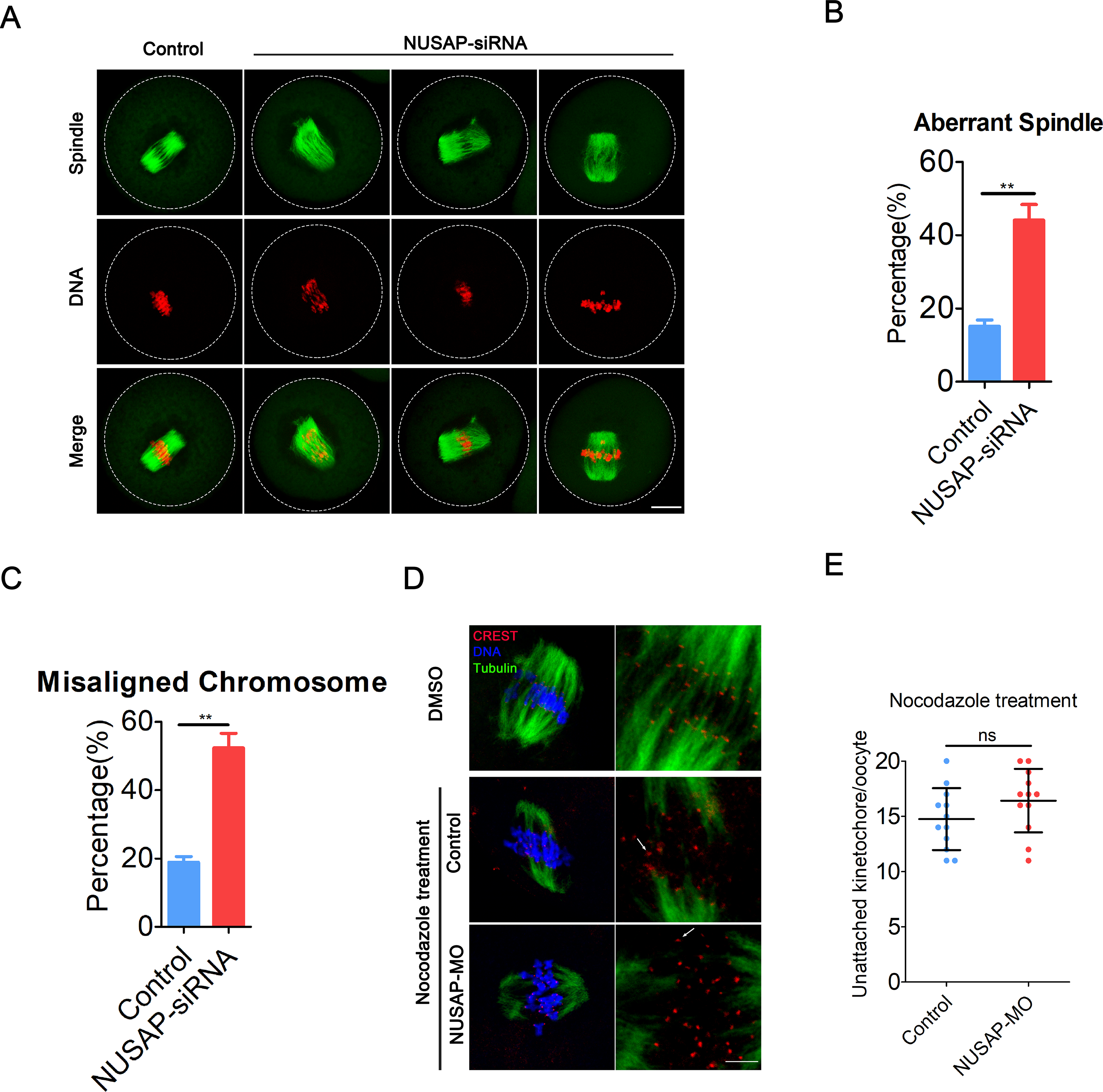


**Figure S4. The effect of NUSAP depletion on spindle assembly, chromosome alignment, and K-MT attachment in mouse oocytes.** (A) Representative images of spindle morphologies and chromosome alignment in control and NUSAP-siRNA oocytes. At 6 h post-GVBD, oocytes were fixed and immunostained for α-tubulin and DNA (PI). Scale bar, 20 μm. (B) The rate of abnormal spindles was recorded in control (n = 79) and NUSAP-siRNA (n = 79) oocytes. (C) The rate of misaligned chromosomes was recorded in control (n = 79) and NUSAP-siRNA (n = 79) oocytes. Data were presented as mean percentage (mean ± SEM) of at least three independent biological replicates. (D) Representative images of K-MT attachment in DMSO and nocodazole treatment oocytes (control and NUSAP-MO oocytes). Oocytes injected with the indicated morpholino or not were cultured with either DMSO or 400 nM nocodazole starting from 4 hours post-GVBD. At 6 hours after GVBD, the oocytes were incubated in M2 medium at 4°C for 10 minutes to induce the depolymerization of unstable microtubules. They were then immediately fixed and immunostained for α-tubulin, CREST, and DNA (Hoechst). White arrows indicate nonconnected kinetochores. (E) The number of unattached kinetochores was recorded in control (n = 12) and NUSAP-MO (n = 12) oocytes after nocodazole treatment. ***P* < 0.01.


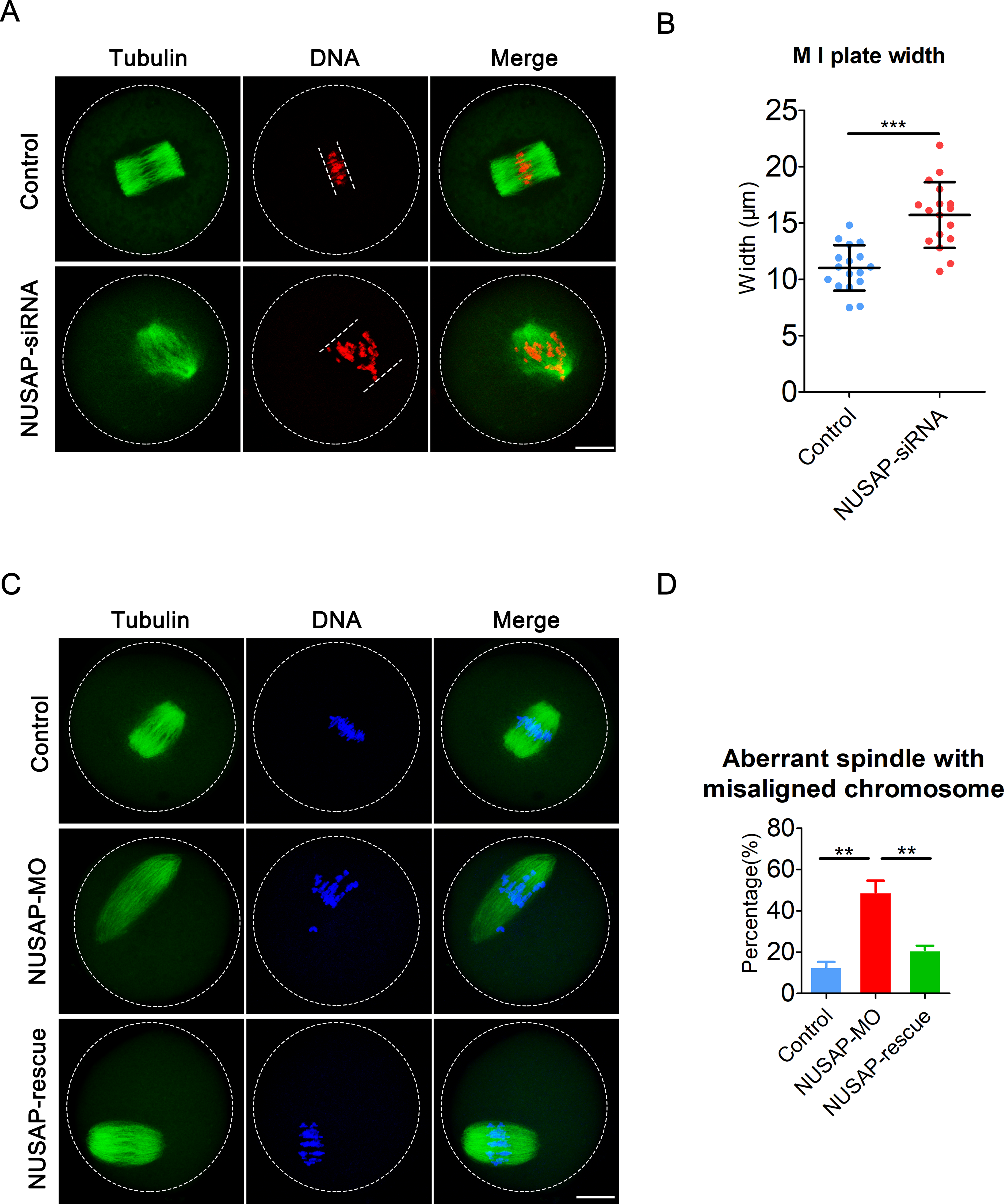


**Figure S5. Effect of NUSAP depletion on the width of M I plate and spindle assembly in mouse oocytes.** (A) Representative images of the width of M I plate in control and NUSAP-siRNA oocytes. At 6 hours after GVBD, oocytes were fixed and immunostained for α-tubulin and DNA (PI). Scale bar, 20 μm. (B) The width of M I plate was measured in control (n = 17) and NUSAP-siRNA (n = 17) oocytes. (C) Representative images of spindle morphologies and chromosome alignment in control, NUSAP-MO, and NUSAP -rescue oocytes. For the rescue experiment, GV oocytes were injected with NUSAP-specific morpholino oligonucleotides and maintained for 20 hours in 50 µM IBMX before being injected with morpholino-resistant NUSAP-mCherry mRNA and maintained for a further 2 hours in 200 µM IBMX to allow time for NUSAP translation. Oocytes were then washed into IBMX-free medium to allow resumption of meiosis. At 6 hours post-GVBD, oocytes were fixed and immunostained for α-tubulin and DNA (Hoechst). Scale bar, 20 μm. (D) The rates of aberrant spindle with misaligned chromosome were recorded in control (n = 39), NUSAP-MO (n = 33), and NUSAP -rescue (n = 33) oocytes. Data of (B) were presented as mean value (mean ± SD) of at least three independent experiments. Data of (D) were presented as mean percentage (mean ± SEM) of at least three independent experiments. ***P < 0.001.


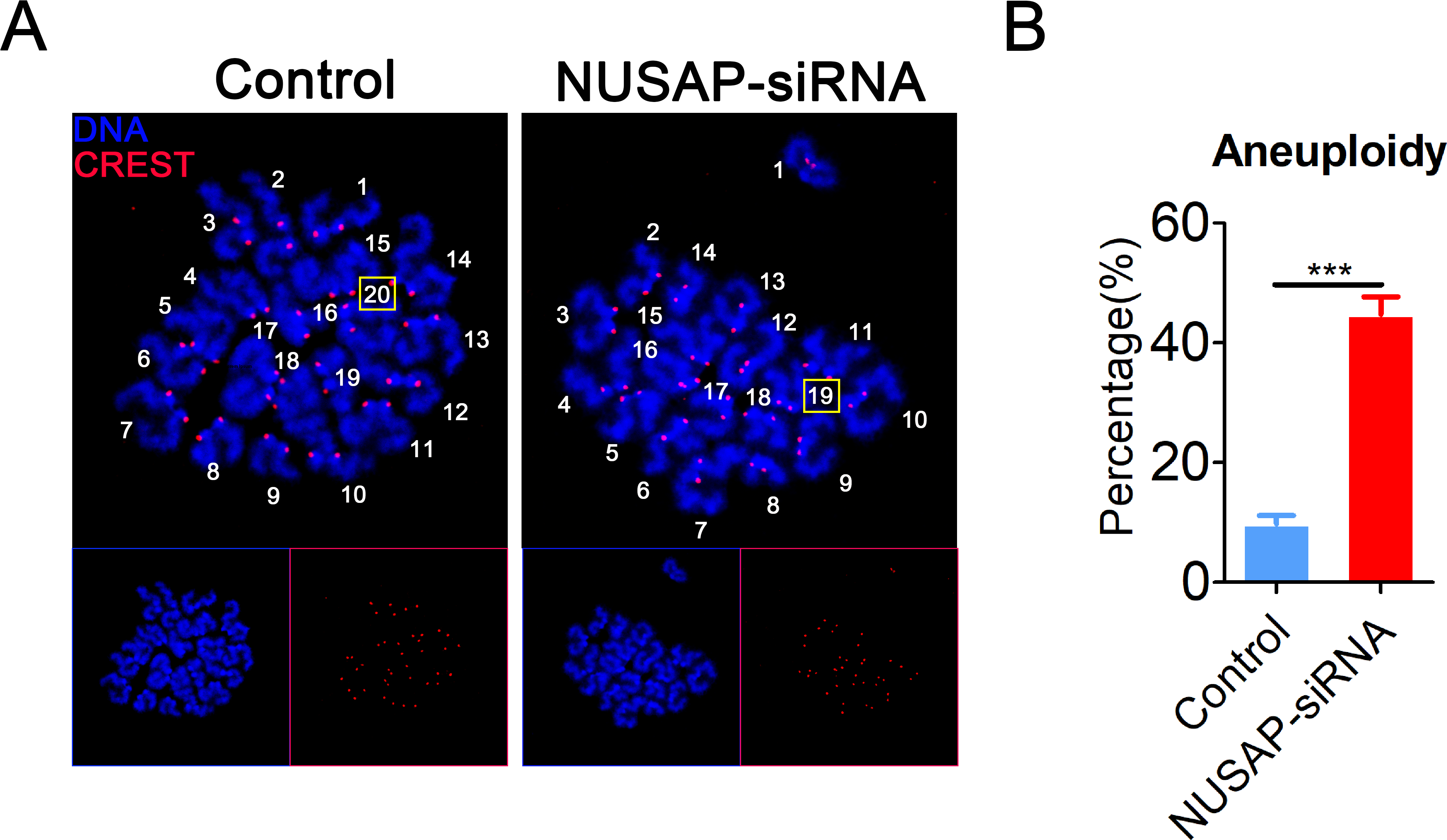


**Figure S6. NUSAP depletion by RNAi** **generates aneuploidy in mouse oocytes.** (A) Representative images of euploid and aneuploid M II eggs. Chromosome spreading was performed to count the number of chromosomes in control and NUSAP-siRNA oocytes at 10 hours after GVBD. The total number of univalents is indicated by the yellow square. (B) The rate of aneuploid eggs was recorded in control (n = 32) and NUSAP-siRNA (n = 29) oocytes. Data of (B) were presented as mean percentage (mean ± SEM) of at least three independent experiments. ****P* < 0.001.


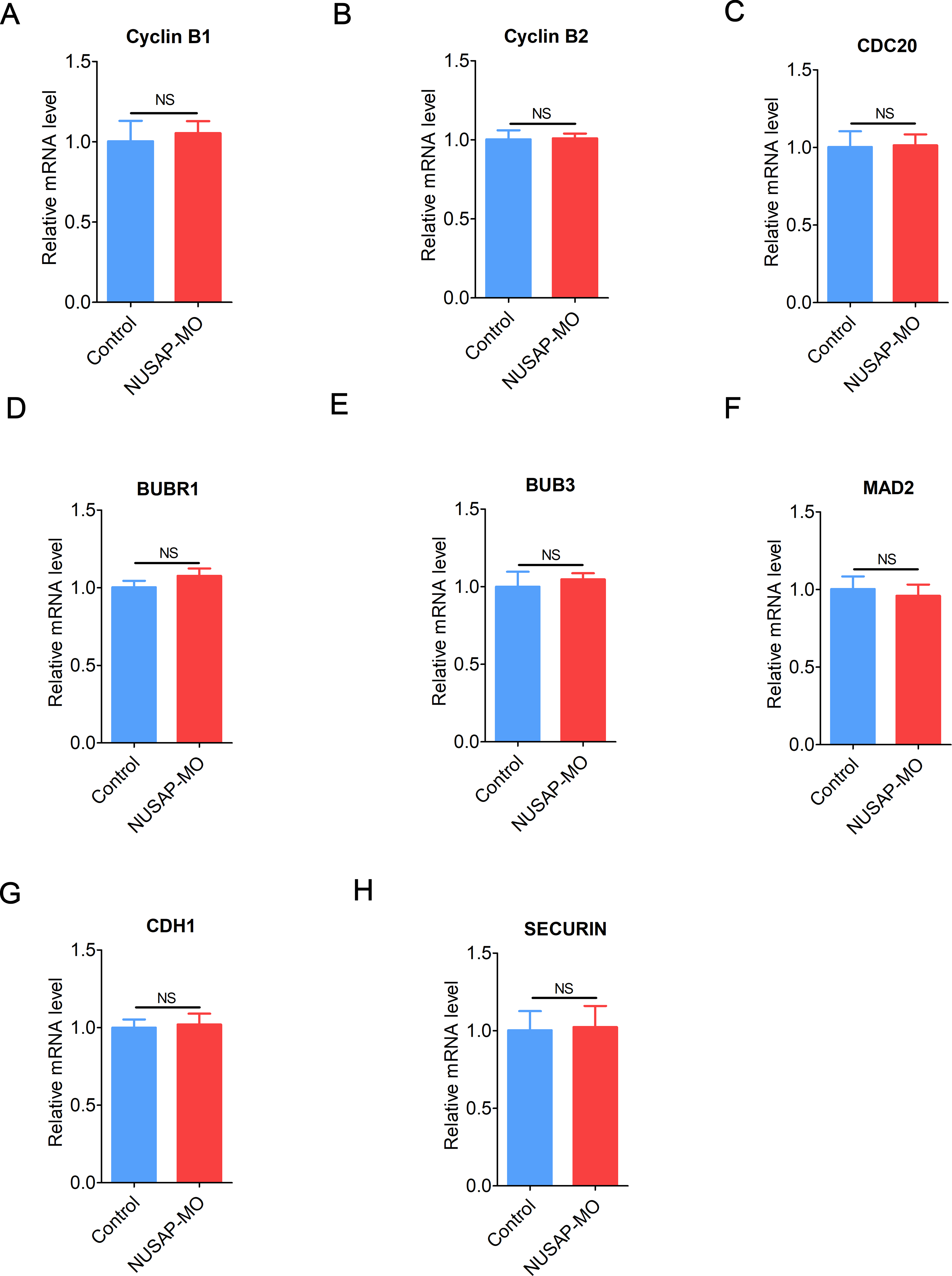


**Figure S7. Effect of NUSAP depletion on the mRNA level of cell cycle proteins in mouse oocytes.** (A-H) mRNA levels of Cyclin B1, Cyclin B2, CDC20, BUBR1, BUB3, MAD2, CDH1, and SECURIN were determined by RT-qPCR in control and NUSAP-MO oocytes at GV stage. NS, not significant.


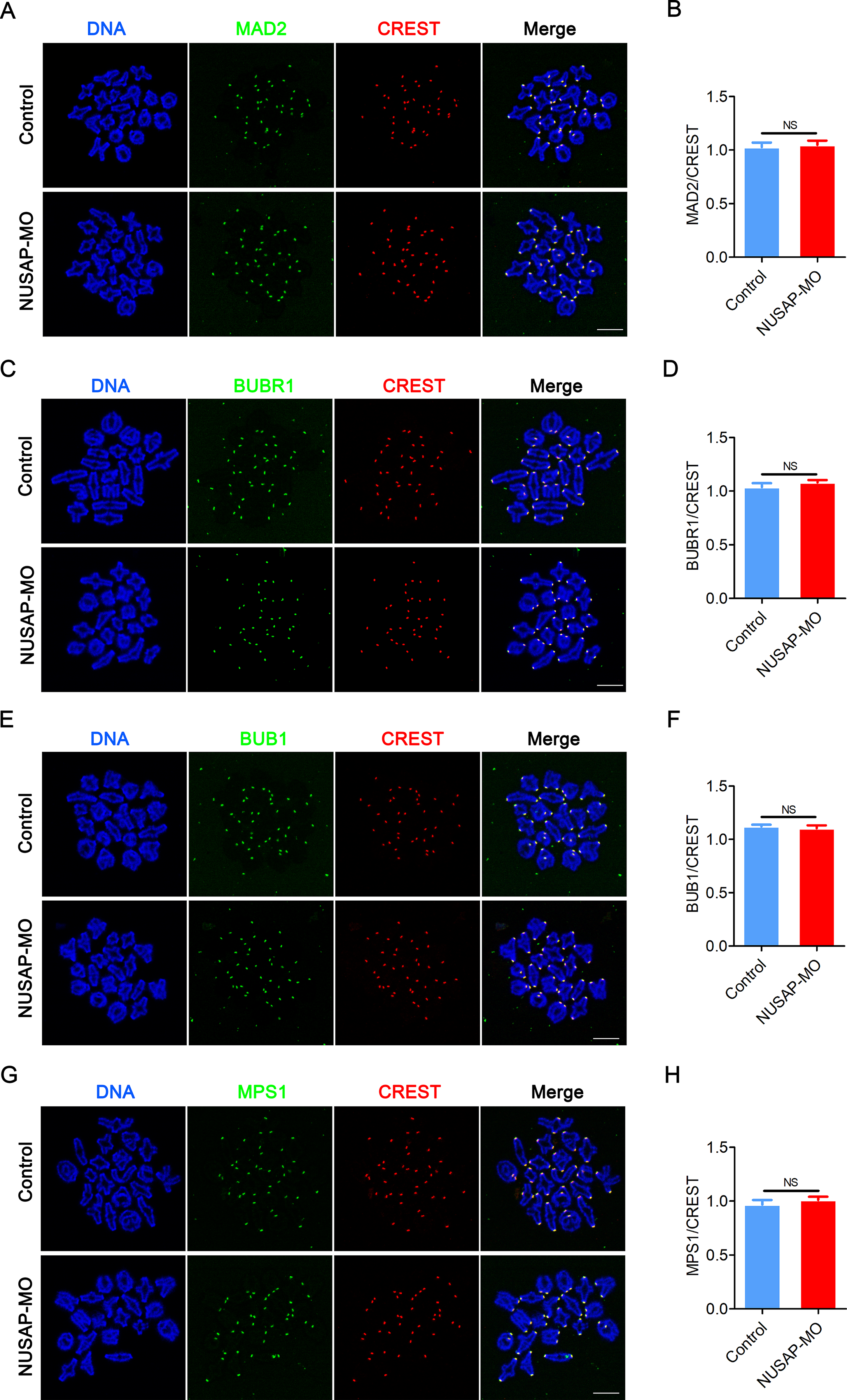


**Figure S8. Effect of NUSAP depletion on the localization of SAC proteins at 5 hours post GVBD.** (A, C, E, G) Representative images of the localization of MAD2, BUBR1, BUB1, and MPS1 in control and NUSAP-MO oocytes, respectively. (B, D, F, H) The relative fluorescence intensities of MAD2, BUBR1, BUB1, and MPS1 compared to CREST were measured in control (n = 200, kinetochores) and NUSAP-depleted (n = 200, kinetochores) oocytes, respectively. The signal intensity was normalized to CREST. NS, not significant.


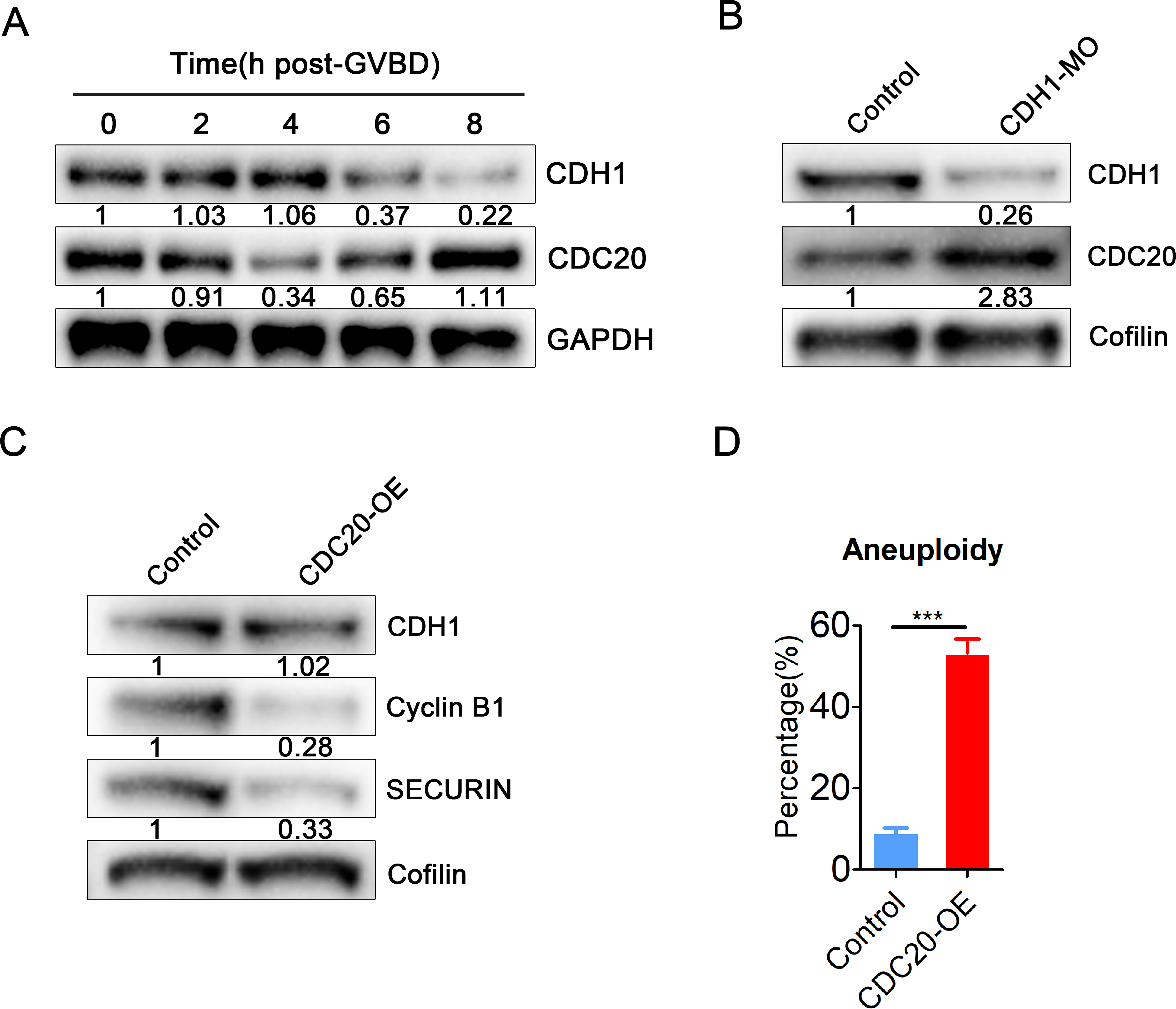


**Figure S9. The effect of CDH1 depletion or CDC20 overexpression on cyclin protein levels or oocyte ploidy.** (A) Western blot analysis of endogenous CDH1 and CDC20 protein levels at the times indicated after GVBD. Assays were performed on the same gel to analyze the levels of CDH1 and CDC20 during the time course of 0, 2, 4, 6, and 8 hours after GVBD. (B) Protein levels of CDC20 were assessed by immunoblots in control and CDH1-MO oocytes at 6 hours post-GVBD. The blots were probed with CDH1, CDC20, and Cofilin antibodies. (C)Protein levels of CDH1, Cyclin B1, and SECURIN in control and CDC20-overexpressed (CDC20-OE) oocytes at 6 hours post-GVBD. Inject CDC20-mRNA at 2 hours post-GVBD, and collect samples for WB at 6 hours post-GVBD. The blots were probed with CDH1, Cyclin B1, SECURIN, and Cofilin antibodies. (D) The rates of aneuploid eggs were recorded in control (n = 34) and CDC20-OE (n = 28) oocytes. Data were presented as mean percentage (mean ± SEM) of at least three independent experiments. ****P* < 0.001.


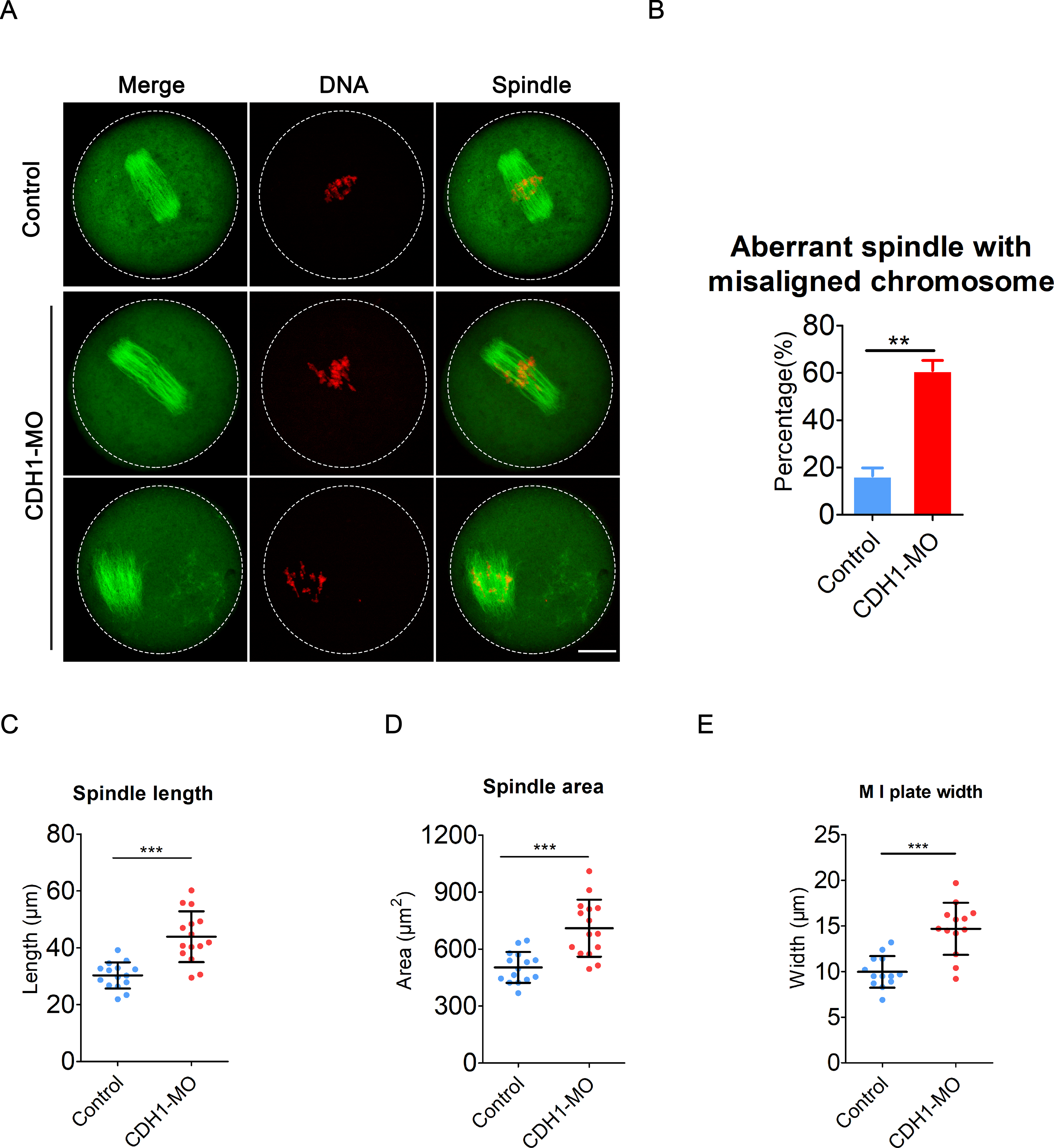


**Figure S10. CDH1 is indispensable for meiotic spindle assembly and chromosome misalignment.** (A) Representative images of spindle morphologies and chromosome alignment in control and CDH1-MO oocytes. At 6 hours post-GVBD, oocytes were fixed and immunostained for α-tubulin and DNA (PI). Scale bar, 20 μm. (B) The rates of aberrant spindle with misaligned chromosomes were recorded in control (n = 55) and CDH1-MO (n = 52) oocytes. (C-D) The spindle length and area were measured in control (n = 15) and CDH1-MO (n = 15) oocytes at 6 hours post-GVBD. (E) The width of M I plate was measured in control (n = 13) and CDH1-MO (n = 13) oocytes. Data were presented as mean percentage (mean ± SEM) or mean value (mean ± SD) of at least three independent biological replicates. ***P* < 0.01, ****P* < 0.001.
